# Effects of *Alhagi camelorum* Fisch polysaccharide from different regions on growth performance and gastrointestinal microbiota of sheep lambs

**DOI:** 10.3389/fphar.2024.1379394

**Published:** 2024-04-30

**Authors:** Zulikeyan Manafu, Zhenping Zhang, Xieraili Malajiang, Saifuding Abula, Qingyong Guo, Yi Wu, Adelijaing Wusiman, Batur Bake

**Affiliations:** ^1^ College of Grassland Science, Xinjiang Agricultural University, Urumqi, China; ^2^ Xinjiang Key Laboratory of New Drug Study and Creation for Herbivorous Animal, College of Veterinary Medicine, Xinjiang Agricultural University, Urumqi, China; ^3^ College of Veterinary Medicine, Nanjing Agricultural University, Nanjing, China

**Keywords:** Alhagi camelorum Fischa polysaccharides, growth performance, intestinal immunity, gastrointestinal microbiota, sheep lambs

## Abstract

Polysaccharides derived from *Alhagi camelorum* Fisch possess diverse activities, making them a potential prebiotic candidates for enhancing lamb health. This study investigated the immunomodulatory effects of *Alhagi camelorum* Fisch polysaccharides from Aksu (AK) and Shanshan (SS) regions on sheep lambs. The results showed that sheep lambs in the SS group exhibited significantly increased (*p* < 0.05) average daily gain, levels of growth hormone (GH), insulin (INS), IgA and IgM, and cytokines IL-4, IL-10, IL-17, TNF-α and IFN-γ compared to those in the control check (CK) group. Moreover, the SS treatment significantly increased the diversity and abundance of beneficial bacteria, while concurrently diminishing the prevalence of harmful bacteria. Additionally, it modulated various metabolic pathways, promoted lamb growth, improved immunity, reduced the risk of gastrointestinal disease and improved the composition of gastrointestinal microbiota. In summary, our findings highlight the potential of SS treatment in enhancing gastrointestinal health of sheep lambs by improving intestinal function, immunity, and gut microbiome. Consequently, these results suggest that *Alhagi camelorum* Fisch polysaccharides derived from Shanshan regions holds promising potential as a valuable intervention for optimizing growth performance in sheep lambs.

## 1 Introduction

The rapid and intensive development of animal farming has led to an increasing presence of pathogenic factors in the production process of juvenile animal (Wang et al., 2020). Environmental pathogens, weak female animals, and malnutrition can adversely affect the growth and development of sheep lambs, impair immune function, and predispose them to various diseases, potentially resulting in mortality (Wang et al., 2021). While antibiotics, vaccines, chemical drugs, and immune stimulants have been widely used in livestock farming with significant effectiveness; their excessive use has resulted in numerous side effects. For instance, the overuse of antibiotics in farming can contribute to antibiotic resistance, disrupt the microbial environment, and lead to the presence of drug residues (Bollen et al., 2023). Therefore, there is an urgent need for a novel feed additive in domestic animal farming that is safe, efficient, without drug residues, and possesses immune-enhancing capabilities to ensure sustainable growth within the livestock industry.

Polysaccharides derived from natural plants have gained significant attention in recent years as key bioactive components in traditional Chinese medicine, owing to their remarkable pharmacological effects (Xue et al., 2023). These plant-derived polysaccharides are now recognized as beneficial nutrients that can promote the activation of immune cells in the intestinal lymph nodes and enhance the secretion of cytokines and antibodies in the lamina propria. Through multifaceted mechanisms, they have ability to improve the immune competence of the intestinal environment, while avoiding harm to the barrier itself (Jiang et al., 2019). According to available literature, the addition of *Astragalus* polysaccharide to the diet of Tibetan lambs has shown a significant increase in overall antioxidant capacity and activities of total superoxide dismutase. Furthermore, it has been observed that this supplementation leads to an elevation in immunoglobulin M (IgM) levels and an augmentation in lymphocyte counts within the bloodstream (Zhong et al., 2012). Adding fermented wheat bran polysaccharides in lamb diet can promote average daily gain, lgG antibody and the contents of IL-1β and IL-10 cytokines, thereby enhancing growth performance and immune function of sheep lambs (Wang et al., 2020). Furthermore, supplementation with Moringa oleifera leaf polysaccharide can improve host health by regulating intestinal mucosal immunity and the ecology of intestinal microorganisms (Zhao et al., 2023). Therefore, these findings suggest that dietary supplements containing polysaccharides offer potential for enhancing intestinal immunity, maintaining microbial balance, and ultimately improving host health.

*Alhagi camelorum* Fisch, a leguminous plant, is a multi-branched semi-shrub commonly found in arid regions of Central Asia, particularly in northwest China, northern India, Kazakhstan, Afghanistan, Iran, Pakistan, Iraq, Mongolia, Syria, and other areas. *Alhagi camelorum* Fisch in China are mainly distributed in Shanshan and Aksu of Xinjiang. (Srivastava et al., 2014). This plant is a valuable traditional medicine known for its efficacy in treating conditions such as abdominal distension, abdominal pain, oral ulcers, and hemoptysis (Li et al., 2018). The primary chemical components of *Alhagi camelorum* Fisch plant include polysaccharides, flavonoids, alkaloids, sterols, fats, and amino acids, among others (Sha et al., 2021). Studies have indicated that *Alhagi camelorum* Fisch polysaccharides (AP) can stimulate the release of TNF-α, IL-1β, and IL-12 cytokine in RAW264.7 macrophages. Moreover, these polysaccharides have shown potential to elevate the circulating IL-2 and IL-10 contents in mice experiencing immunosuppression (Zhao et al., 2020). The Alhagi honey polysaccharide secreted from *Alhagi camelorum* Fisch can be used as a dietary additive to enhance the intestinal immune function of healthy mice, regulate the structure of microbial flora, improve the intestinal immune function of immunosuppressed mice and repair intestinal barrier damage (Cai et al., 2021a; Cai et al., 2021b). Feeding Alhagi honey polysaccharide can promote the growth performance and immune organ index of chicks, and improve the structure of intestinal microflora (Cai et al., 2022a). However, there is currently limited research on regional variations in the biological function of AP and its effects on immune function and gastrointestinal flora in sheep lambs.

Therefore, in this study, sheep lambs were orally administered AP derived from Shanshan and Aksu regions. The effects of the polysaccharide on growth performance of sheep lambs were evaluated by measuring changes in average daily body weight gain, body length, and levels of growth-related hormones. The effects of the polysaccharide on immune function of sheep lambs were determined by changes in antibody levels, cytokines and gastrointestinal structure. Finally, by analyzing the effects of the polysaccharide on lamb gastrointestinal microorganisms, a detailed evaluation of the polysaccharide’s effects on growth promotion, immune enhancement and gastrointestinal healthcare was conducted. This research aims to screen out AP that are the best in promoting lamb growth, enhancing intestinal immunity, and regulating gastrointestinal flora, and thus provide alternative antibiotics materials that support the healthy growth of lambs.

## 2 Materials and methods

### 2.1 Materials

*Alhagi camelorum* Fisch were collected from Sanhe Town, Awati County, Aksu City (longitude: 80°13′43.79″, latitude: 40°16′59.52″), and Dikan Town, Shanshan County, Turpan City (longitude: 89°47′20.82″, latitude: 42°35′43.11″).

### 2.2 Animal experiments

The experimental group comprised of 30 sheep lambs, approximately 5 days old, with an average weight of 2.796 ± 0.267 kg. All animal experiments were conducted at the Puhui Township Farm in Korla City. The experiment (approval number: 2022016) received approval from Xinjiang Agricultural University IACUC to ensure compliance with relevant ethical regulations for animal experimentation and research. On day 28, six sheep lambs in each group were randomly selected for euthanasia by neck bleeding, and their intestinal and rumen tissues were collected for subsequent experiments.

### 2.3 Extraction of AP

The AP were obtained from *Alhagi camelorum* Fisch through a series of process including water extraction, alcohol precipitation, and purification (Alhidary and Abdelrahman, 2016). Our previous studies have shown that *Alhagi camelorum* Fisch has the highest polysaccharide content in September. Therefore, specimens of *Alhagi camelorum* Fisch, including leaves, stems, and spines, were collected from Aksu and Shanshan areas in September. The collected specimens were dried in electric air circulating dryer. Next, the dehydrated specimens were boiled and subjected to extraction. The resulting extract was concentrated to achieve a 1 g/mL concentration. Subsequently, anhydrous ethanol was added to the concentrated extract until it reached at a concentration of 80% ethanol. The solution was then cooled overnight at 4 °C, and the precipitate was collected and dried to evaporate the excess ethanol. After the precipitate was completely dried, the polysaccharides SS and AK were obtained. The polysaccharide content, glucuronate content and protein content of SS and AK were detected by phenol-sulfuric acid method, carbazole sulfate method and BCA kit, respectively. The polysaccharide content of SS and AK was found to be 32.9% and 27.3% respectively, while the glucuronate content was 12.4% for SS and 8.5% for AK. The protein content was measured to be 3.1% for SS and 3.7% for AK.

### 2.4 Experimental design and management protocols

**FIGURE 1:**
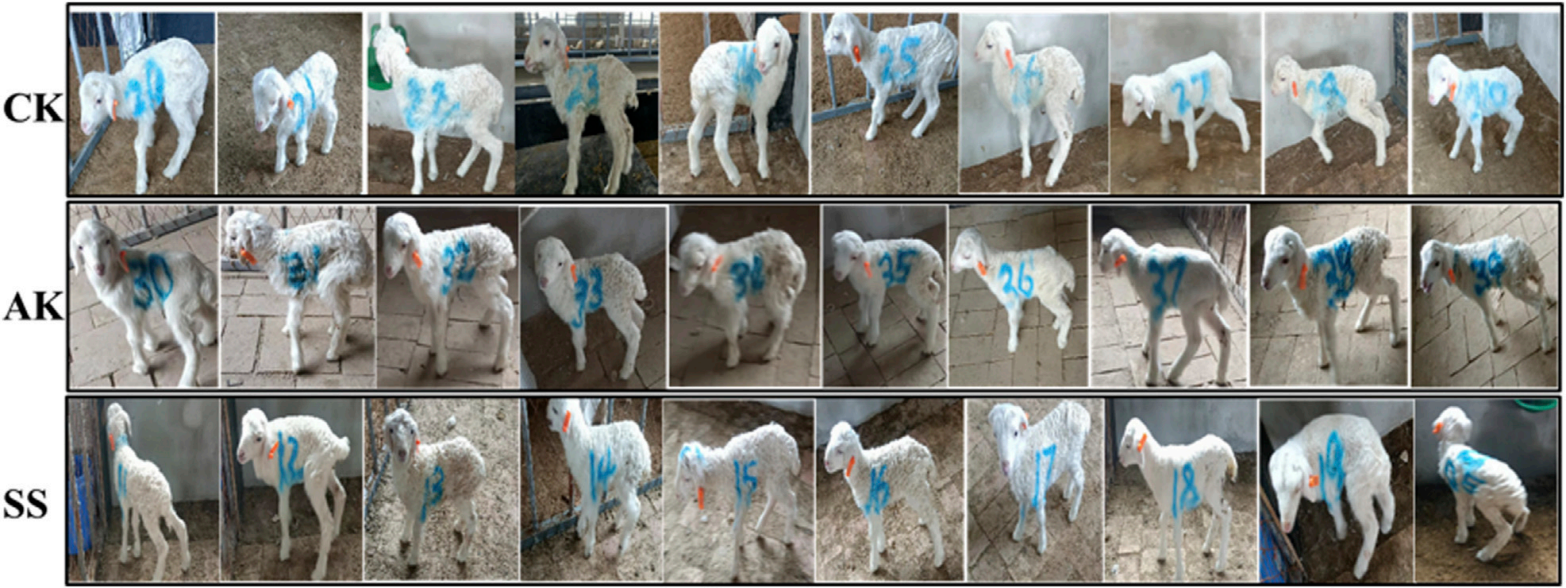
Group of sheep lambs.

A total of thirty sheep lambs, each with an initial weight of 5 days old, were allocated into three groups according to their individual weights. Each group consisted of ten sheep lambs. These groups were as follows: AP from Aksu (AK, 1.5 g/kg AK aqueous solution daily), AP from Aksu Shanshan (SS, 1.5 g/kg AK aqueous solution daily) and control group (CK, administration of equal volume of normal saline daily). During the experimental period, consistent feeding methods, management practices, and environmental conditions were maintained across all groups. Feed and water levels for each ewe were uniformly managed throughout the experiment period, and sheep lambs were allowed to feed freely from their mothers. The sheep lambs remained with the ewes for 20 days, feeding and nursing without restrictions. Specifically, they stayed with ewes for 17 days after the start of the experiment, feeding and nursing freely. After 17 days, the sheep lambs were managed, still having access to free feeding and drinking, but the duration of lactation was limited to 10 h per day Figure 1.

### 2.5 Measurement of growth performance of sheep lambs

The body weight of each lamb was measured weekly throughout the entire experiment. Measurements were conducted to assess the body height, bust length, and pipe circumference length at the beginning and end of the experiment. Various metrics, including average daily gain, body height, bust length, and pipe circumference length were calculated. Additionally, blood samples were collected from sheep lambs’ jugular vein following 28 days of feeding.

### 2.6 Determination of growth factor, immunoglobulin and cytokines contents of sheep lambs

On day 28, blood samples were obtained and serum was separated to measure the levels of growth hormone (GH), insulin-like growth factor-1 (IGF-1), and insulin (INS) using a ELISA kit (Jiancheng Bioengineering Co., Ltd, Nanjing, China.). Total serum IgM was measured using ELISA kits (FanKew Biotech Co., Ltd, Shanghai, China). To measure the total intestinal sIgA and cytokines, a 2 cm segment of the small intestine was obtained and homogenized with 2 mL of PBS. The resulting supernatant was then collected by centrifugation at 3,000 rpm for 15 min. Total sIgA, IgM, IL-4, IL-10, IL-10, IL-17, TNF-α, and IFN-γ contents were measured using ELISA kits (FanKew Biotech Co., Ltd, Shanghai, China).

### 2.7 Histopathological examination of rumen and intestine of sheep lambs

On the 28th day, tissue specimens from the rumen, duodenum, jejunum, and ileum were collected and immersed in a 4% paraformaldehyde solution to facilitate fixation. These tissue samples were then processed for staining by embedding them in paraffin wax blocks, cutting them into sections with a thickness of 3–5 μm, and staining them with hematoxylin and eosin (HE). Subsequently, a histopathological examination was conducted to observe alterations in their histological morphology.

### 2.8 Determination of rumen and rectum microbiota in sheep lambs

On day 28, six sheep lambs were randomly selected from each group to obtain samples of rumen contents and rectal feces. The collected specimens were stored in containers containing liquid nitrogen and transported to the laboratory for DNA isolation. The quantity of DNA was assessed utilizing a nanodrop, followed by purification, PCR amplification, and fluorescence quantification procedures. Subsequently, a computer-based sequencing was performed to prepare a sequencing library. The analysis of intestinal and rumen microorganisms was carried out by Nomi Biological Company.

### 2.9 Statistical analysis

The statistical software SPSS 24.0 was employed to perform one-way analysis of variance (ANOVA) to identify significant differences. Graphs were generated using GraphPad Prism 5. All presented results represent the average values and standard errors of the mean (SEM). Different letters in the superscript bars indicate significant difference (*p* < 0.05).

## 3 Results and analysis

### 3.1 Effects of AP from different regions on growth performance of sheep lambs

The impact of AP from various regions on the growth performance of sheep lambs are summarized in Table 1. No statistically significant differences were observed among all experimental groups compared to the control group, with regards to initial body weight, initial body height, initial bust length, and initial pipe circumference length (*p* > 0.05). However, on day 28 after AP administration, the final body weight, average daily gain, final bust length and final pipe circumference length of the SS group were significantly higher than that of the CK group (*p* < 0.05).

**TABLE 1:** Effects of AP from different regions on growth performance and body size of sheep lambs.

Item	CK	AK	SS	Sem	*p*-value
Initial body weight (kg)	2.72	2.73	2.72	0.07	0.999
Final body weight (kg)	4.27^b^	4.733^ab^	5.43^a^	0.21	0.027
Average daily gain (g/d)	55^b^	74.28^ab^	97.62^a^	6.84	0.008
Initial body height (cm)	34.55	35.66	34.75	0.67	0.035
Final body height (cm)	38.17^a^	41.16^a^	43.16^a^	0.63	0.000
Initial bust length (cm)	32.63	31.53	32.03	0.45	0.345
Final bust length (cm)	35.33^b^	37.08^ab^	40.95^a^	1	0.02
Initial pipe circumference length (cm)	4.71	4.72	4.68	0.08	0.935
Final pipe circumference length (cm)	5.17^b^	5.63^a^	6^a^	0.108	0.000

Data are presented as mean ± standard deviation (n = 6). Superscripts with different letters (a, b) indicate a significant difference (*p* < 0.05); superscripts with a same letter (a, ab) indicate trends were considered, but the difference is not significant (0.05 < *p* < 0.10).

### 3.2 Effects of AP from different regions on serum growth factor of sheep lambs

**FIGURE 2:**
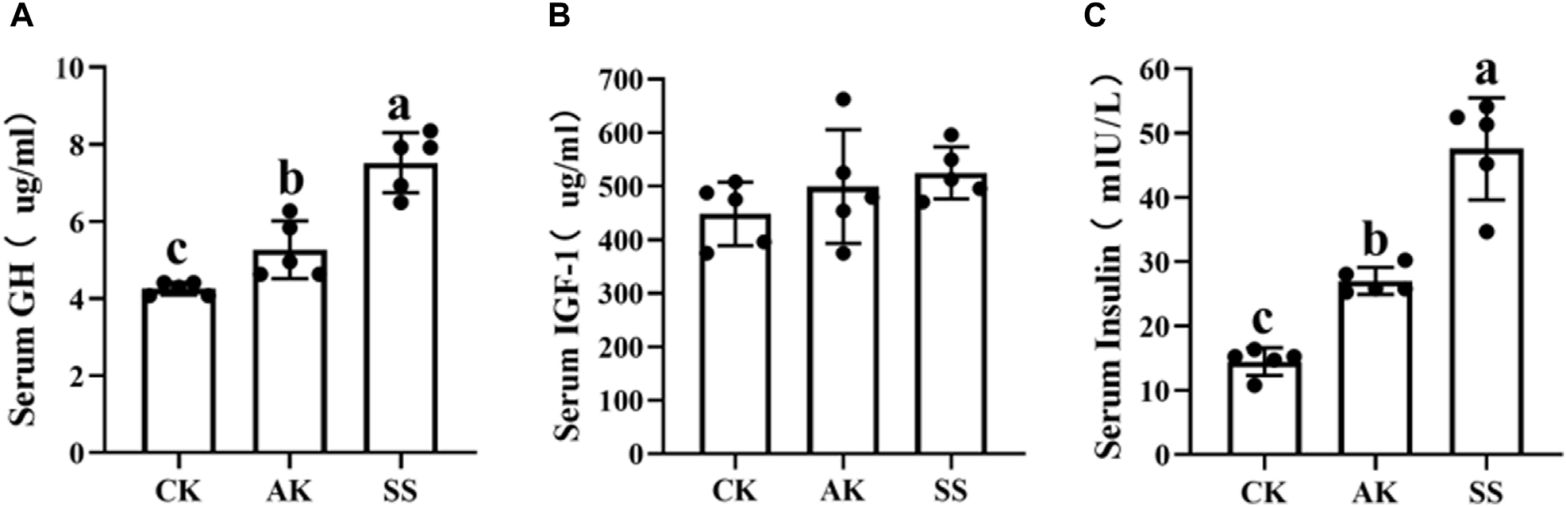
Effects of AP from different regions on serum growth factors of sheep lambs. **(A)** Serum GH Content. **(B)** Serum IGF-1 Content. **(C)** Serum INS Content. Data are presented as mean ± standard deviation (n = 5). Bars with different superscripts **(a–c)** indicate significant differences (*p* < 0.05).

To investigate the impact of AP from different region on growth-related hormone levels in sheep lambs, we analyzed the serum concentrations of GH, INS, and IGF-1. Figure 2A-C demonstrate that the SS group exhibited significantly elevated levels of GH and INS compared to both the AK and CK groups (*p* < 0.05). However, no significant differences were observed in the IGF-1 level among all experimental groups (*p* > 0.05).

### 3.3 Effects of AP from different regions on the immunoglobulin content in sheep lambs

**FIGURE 3:**
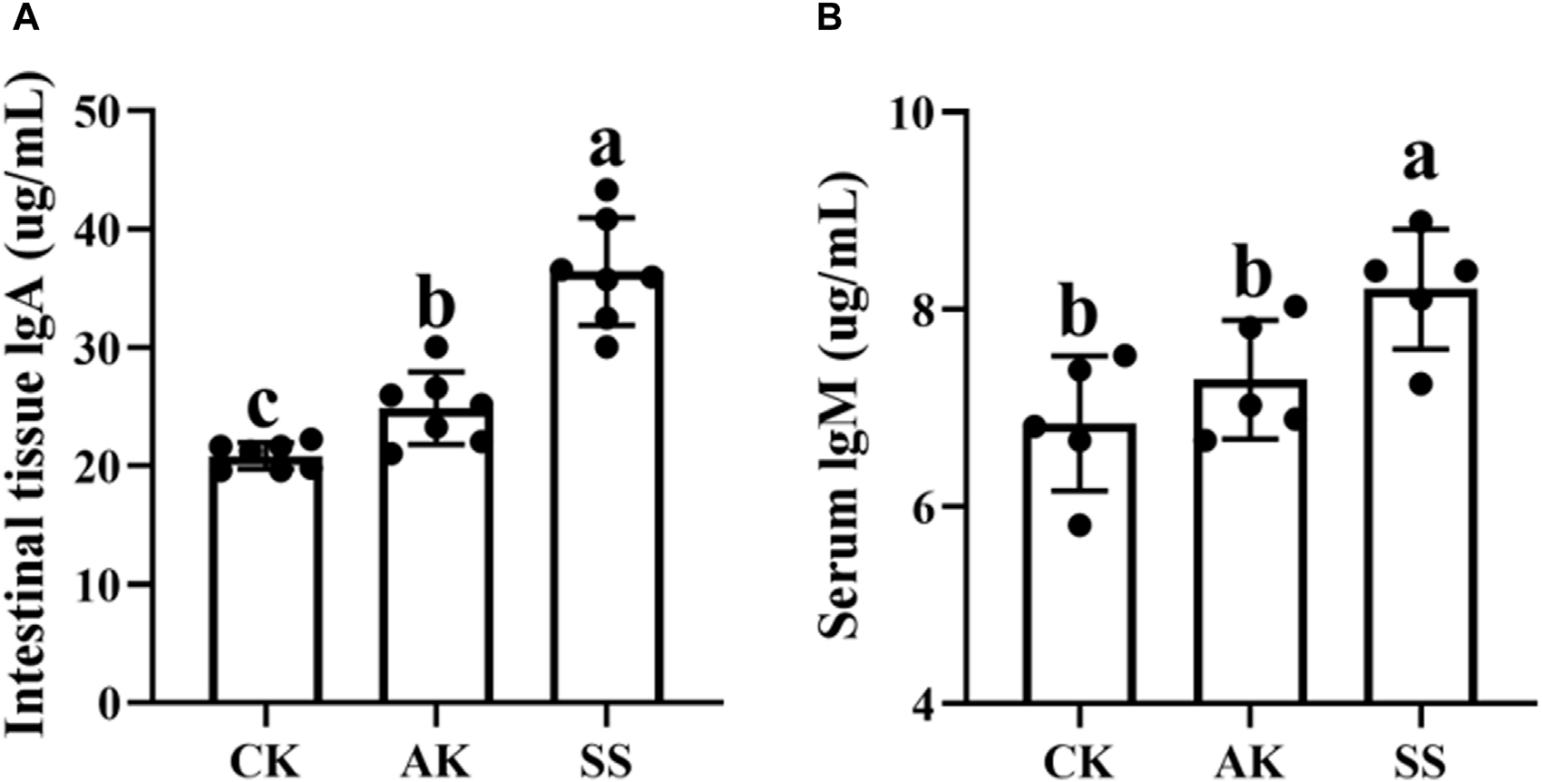
Effects of AP from different regions on intestinal immunoglobulin content of sheep lambs. **(A)** Intestine IgA content. **(B)** serum IgM content. Data are presented as mean ± standard deviation (n = 7). Bars with different superscripts **(a–c)** indicate significant differences (*p* < 0.05).

To study the effect of AP on sIgA and IgM in sheep lambs, we assessed the content of sIgA in small intestine and IgM in serum. As depicted in Figure 3A, B, the levels of sIgA and IgM were significantly elevated in the SS group compared to both the CK and AK groups (*p* < 0.05). Furthermore, the AK group exhibited a statistically significant increase in sIgA levels compared to the CK group (*p* < 0.05), whereas there was no notable difference observed between the IgM content of both groups (*p* > 0.05).

### 3.4 Effects of AP from different regions on intestinal cytokine levels in sheep lambs

**FIGURE 4:**
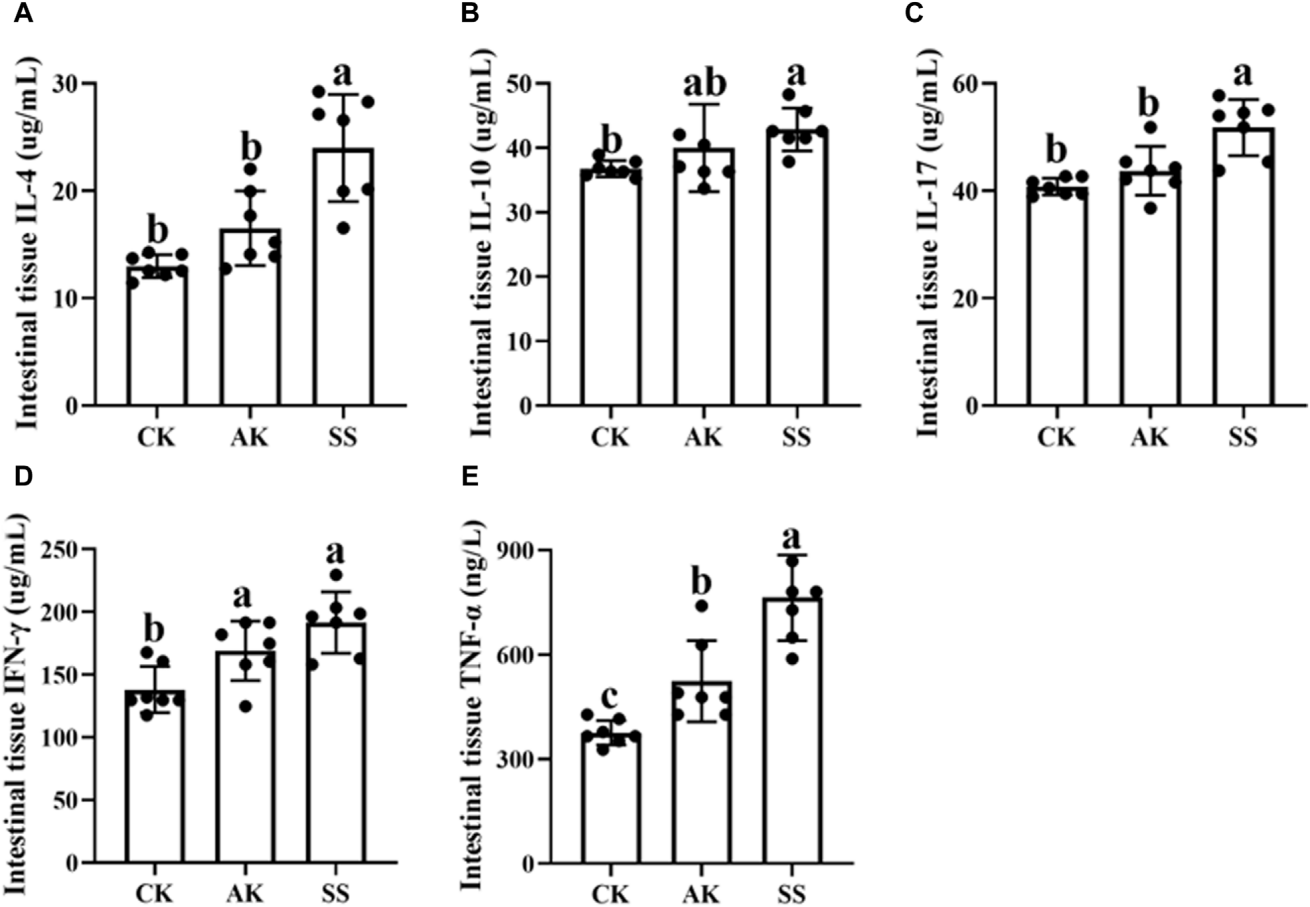
Effects of AP from different regions on intestinal cytokines level of sheep lambs. **(A)** Intestine IL-4 Content. **(B)** Intestine IL-10 Content. **(C)** Intestine IL-17 Content. **(D)** Intestine IFN-γ Content. **(E)** Intestine TNF-α Content. Data are presented as mean ± standard deviation (n = 7). Bars with different superscripts **(a–c)** indicate significant differences (*p* < 0.05).

The effects of AP from different regions on intestinal cytokine content of sheep lambs are shown in Figure 4. The levels of IL-4, IL-10, IL-17, TNF-α, and IFN-γ were significantly elevated in the SS group compared to the CK group (*p* < 0.05). Additionally, the concentrations of IL-4, IL-17, and TNF-α were significantly higher in the SS group than in the AK group (*p* < 0.05). There was a noticeable increase in TNF-α and IFN-γ levels in the AK group compared to the CK group (*p* < 0.05), and no significant differences were detected for other cytokines between these two groups (*p* > 0.05).

### 3.5 Effects of AP from different regions on intestinal and rumen histology in sheep lambs

**FIGURE 5:**
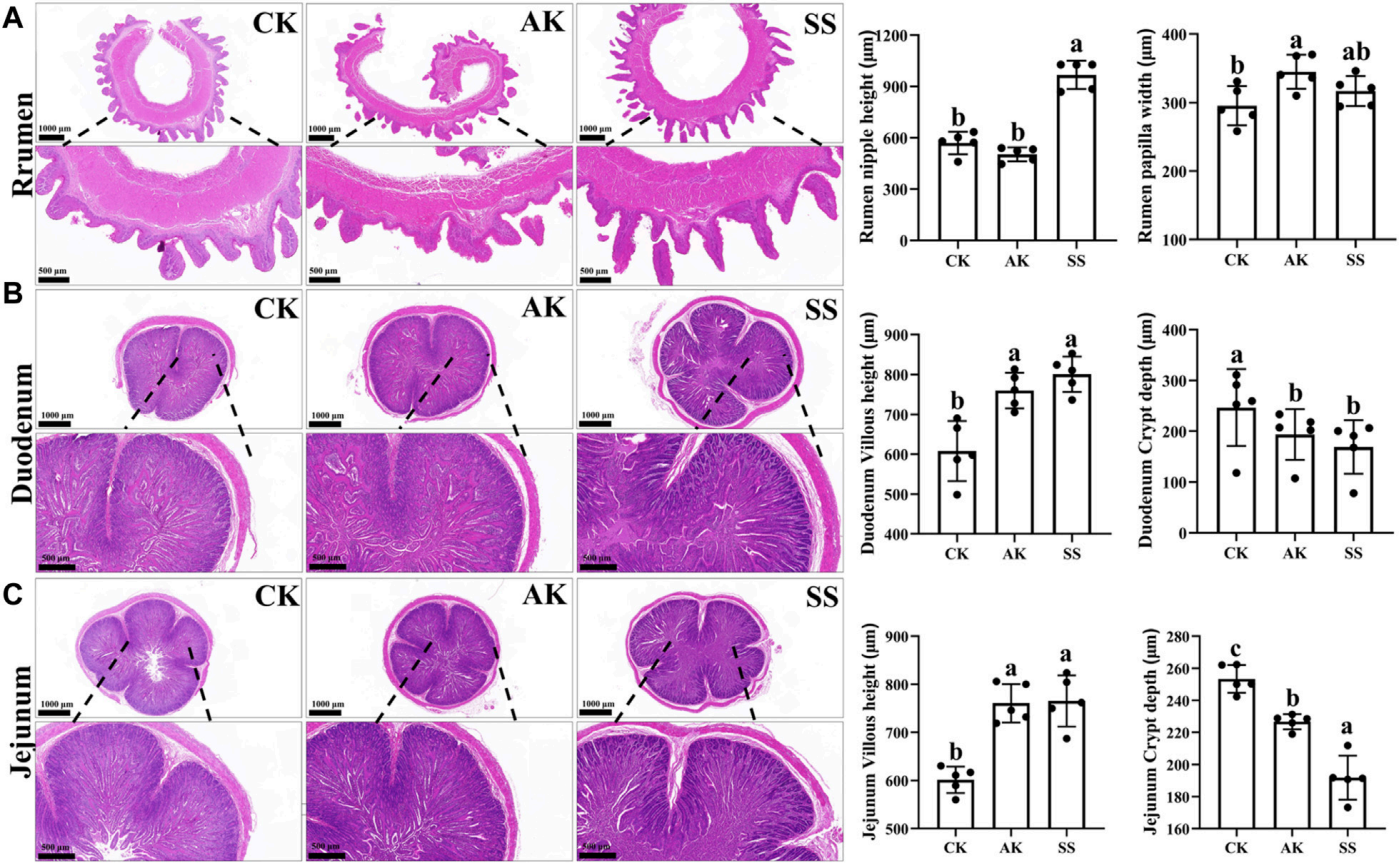
Effects of AP on structural morphology of gut and rumen in sheep lambs. **(A)** Rumen papillary height and width. **(B)** Villus height and crypt depth of duodenum. **(C)** Villus height and width of jejunum. Data are presented as mean ± standard deviation (n = 5). Bars with different superscripts superscripts **(a–c)** indicate significant differences (*p* < 0.05).

No physiological structural abnormalities were observed in the rumen, duodenum and jejunum of CK, AK, and SS groups (Figures 5A–C). As shown in Figure 5A, rumen papillary height in SS group was significantly higher than that in AK and CK groups (*p* < 0.05), while the rumen papillary width was intermediate between the AK and CK groups and did not show significant difference between AK and CK groups (*p* > 0.05). The rumen papillary width in AK group was significantly higher than that in CK group (*p* < 0.05). As depicted in Figures 5B, C, the villous length of the duodenum and jejunum exhibited a significant increase in both the SS group and AK group compared to the CK group (*p* < 0.05). Meanwhile, a significant reduction in crypt depth was detected in both the duodenum and jejunum of the SS and AK groups compared to the CK group (*p* < 0.05).

### 3.6 Effects of AP from different regions on the intestinal and rumen microbiota in sheep lambs

#### 3.6.1 Alph (α) diversity analysis

**FIGURE 6:**
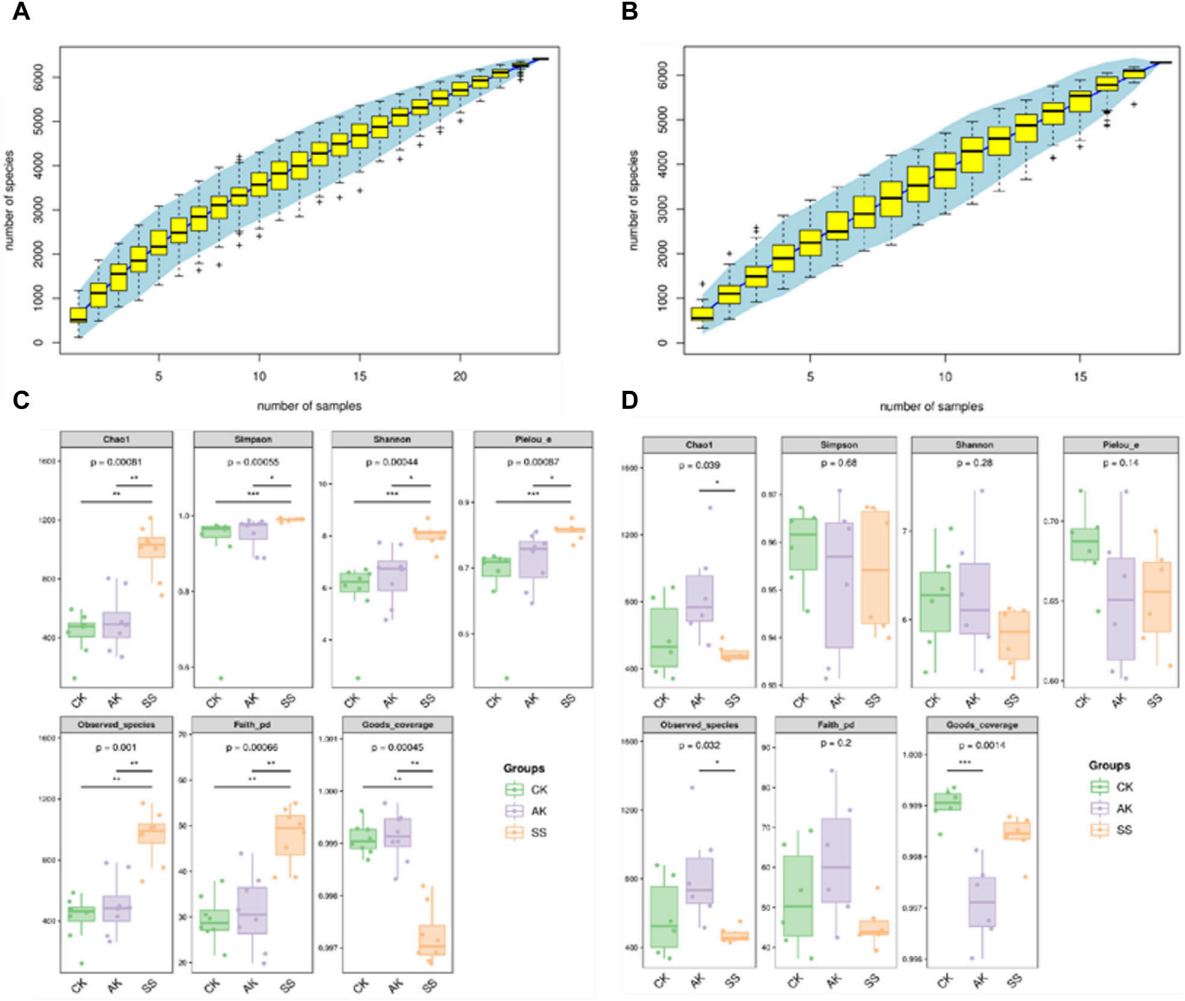
The species accumulation curves and α-diversity analysis of small intestine and rumen flora in sheep lambs. **(A)** Species accumulation curves of intestinal microorganisms, **(B)** Species accumulation curves of rumen microorganism, **(C)** α-diversity analysis of small intestine microorganism, **(D)** α-diversity analysis of rumen microorganism.

Species accumulation curves are used to measure and predict whether the sample size is sufficient and to estimate community richness (Park et al., 2017). As depicted in Figures 6A, B, the stability of the species accumulation curves for intestinal and rumen flora suggests that the sequencing outcomes adequately capture the diversity of samples within each treatment group, ensuring reliable results for subsequent analysis. As illustrated in Figures 6C, D, Goods_coverage surpasses of 0.997 for every group, indicating the completeness and reliability of sample sequencing results across all groups. The diversity of intestinal and rumen flora in sheep lambs was examined, as depicted in Figure 6C. In comparison to the CK group, the AK and SS groups exhibited increased values for intestinal Chao1, Simpson, Shannon, Pielou’s evenness, Observed_species, and Faith’s indexes. Specifically, the SS group displayed significantly higher values for these indexes compared to both the AK and CK groups (*p* < 0.05). However, there was no significant difference observed between the CK and AK groups (*p* > 0.05). According to the data presented in Figure 6D, there was a significant increase in rumen Chao1 and Observed_species indices observed in the AK group compared to the SS group (*p* < 0.05). However, no significant differences were found among all groups for other rumen index indices (*p* > 0.05).

#### 3.6.2 Beta (β) diversity analysis

**FIGURE 7:**
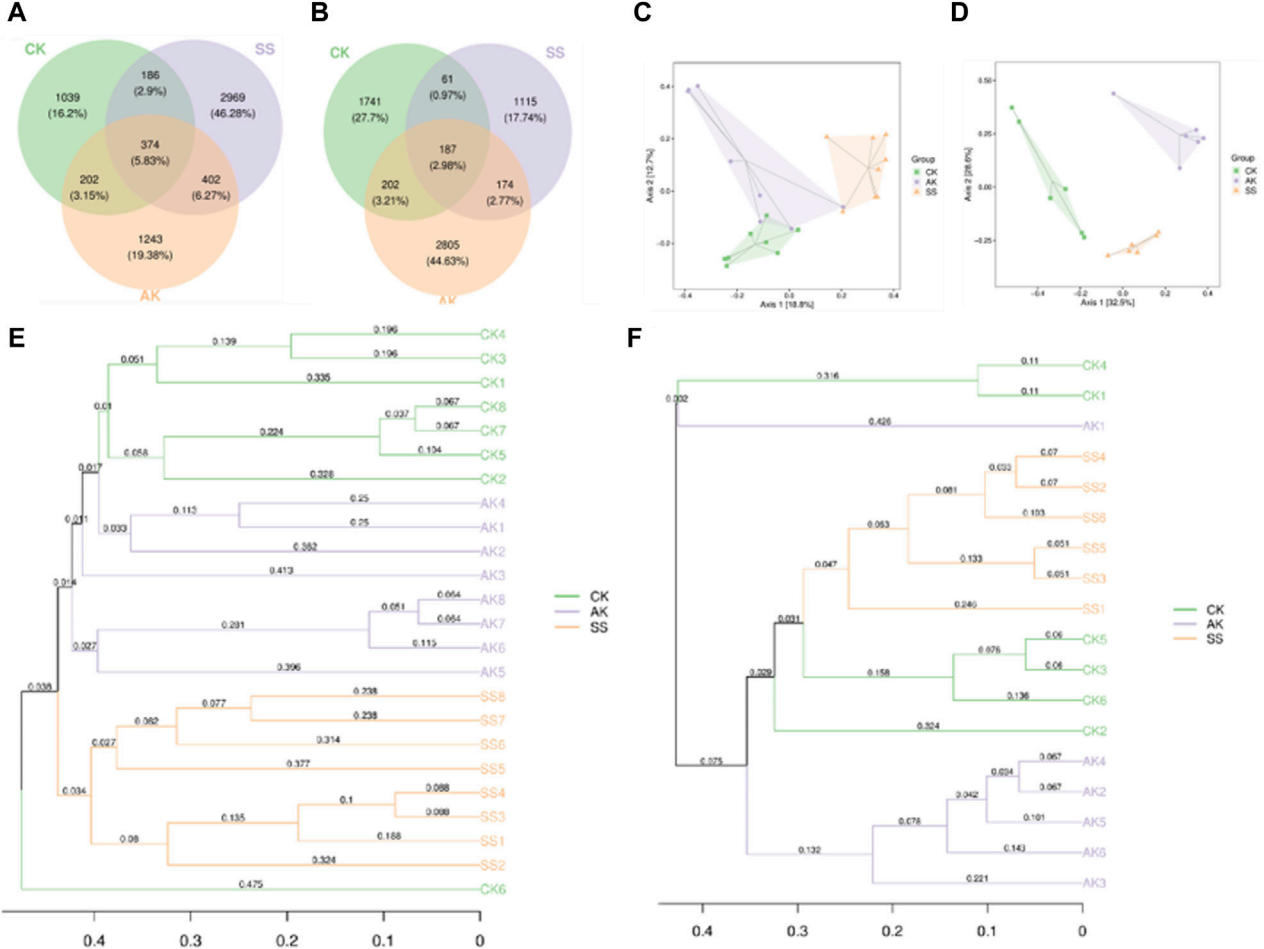
β-Diversity analysis of microorganism. **(A)** Venn diagram of intestine microorganism based on the ASV levels, **(B)** Venn diagram of rumen microorganism based on the ASV levels, **(C)** PCoA analysis of intestine microorganism based on Bray-Crutis, **(D)** PCoA analysis of rumen microorganism based on Bray-Crutis, **(E)** Hierarchical clustering analysis of intestine microorganism based on Bray-Crutis, **(F)** Hierarchical clustering analysis of rumen microorganism based on Bray-Crutis.

Microbial β-diversity analysis is used to explore differences in microbial community composition. As shown in Figure 7A, a total of 5,251 Amplicon Sequence Variants (ASVs) were identified from intestinal samples, of which 5.83% ASVs were common to all three groups. The proportion of ASVs unique to CK, AK and SS groups were 16.2%, 19.38% and 46.28%, respectively. As shown in Figures 7A, B total of 5,661 ASVs were identified in the rumen samples, of which 2.98% ASVs were common to all three groups. The proportion of ASVs unique to CK, AK and SS groups were 27.7%, 44.63% and 17.74%, respectively. The results of microbial β-diversity analysis in the intestinal are shown in Figure 7C, where the CK and SS groups were completely separated, while CK and AK groups overlapped slightly. In the rumen, microbial PCoA analysis results are presented in Figure 7D, where the AK, CK and SS groups were entirely separated. The UPGMA cluster analysis diagram provided additional evidence of distinct clustering between the CK, AK and SS groups, indicating noticeable variations in flora composition among the these groups (Figures 7B,F).

#### 3.6.3 Phylum level analysis

**FIGURE 8:**
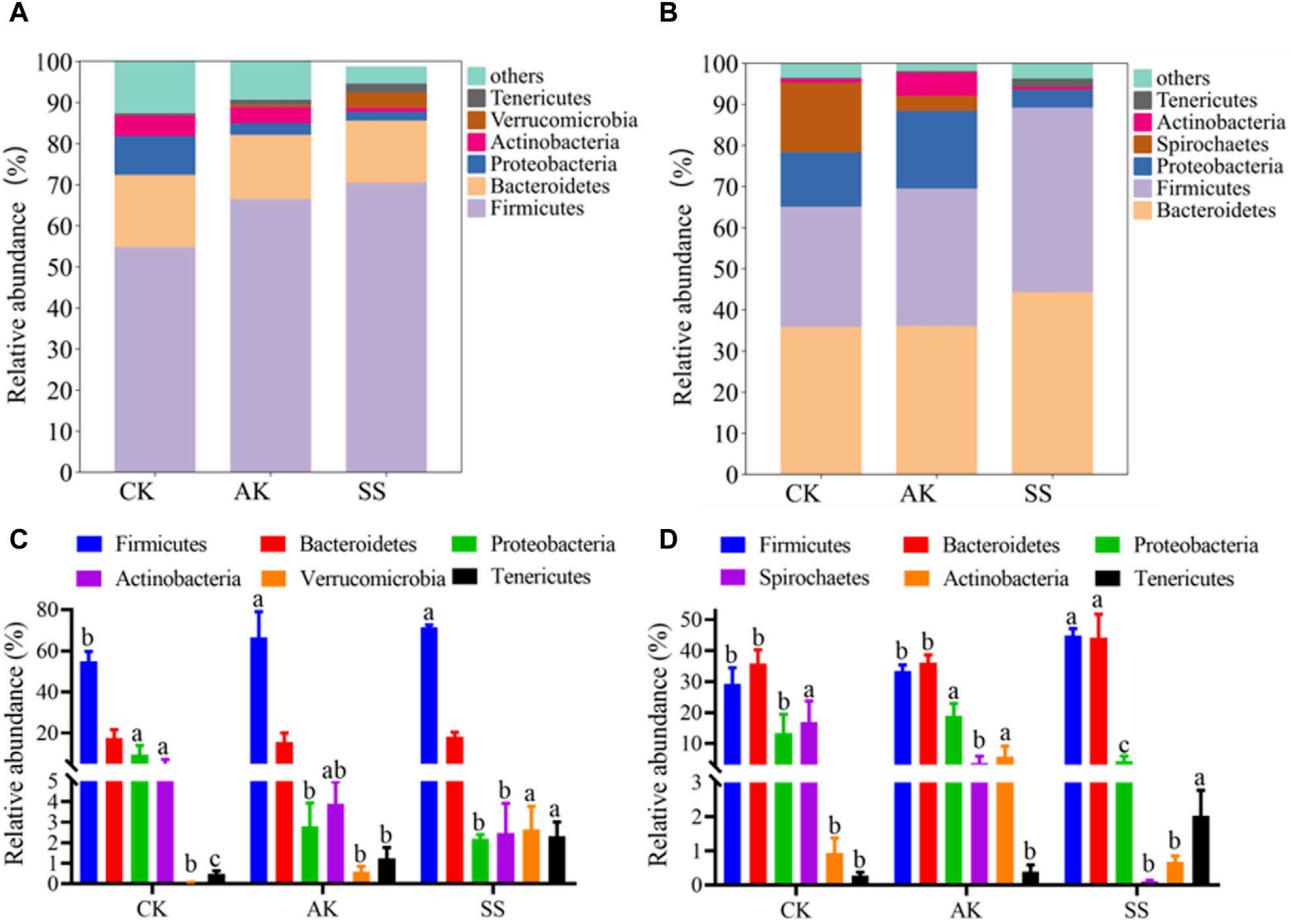
Relative abundance of the predominant microorganism at the phylum level. **(A)** Phylum-level distribution of main microorganisms in intestines, **(B)** Phylum-level distribution of main microorganism in rumen, **(C)** Bar chart illustrating species composition of intestinal microorganisms at the phylum level, **(D)** Bar chart illustrating species composition of rumen microorganisms at the phylum level . Data is presented as mean ± standard deviation (n = 6). Bars with different superscripts **(a–c)** indicate significant differences (*p* < 0.05).

Effects of AP from different regions on the phylum level of microbiome in intestinal and rumen of sheep lambs were shown in Figure 8A, B. At the phylum level, there were similarities in the dominant bacteria detected in both intestines and rumen, including, *Firmicutes*, *Bacteroidetes*, *Proteobacteria*, *Actinobacteria* and *Tenericutes*. As depicted in Figure 8C, D, the SS group exhibited a significantly higher relative abundance of *Firmicutes* in the intestine, rumen *Firmicutes*, and *Tenericutes* compared to the AK and CK groups (*p* < 0.05). However, there was no significant difference observed between the SS group and AK group regarding the abundance of intestine *Firmicutes* (*p* > 0.05). Furthermore, the relative abundance of intestinal *Proteobacteria* was notably lower in the SS group than in the CK group (*p* < 0.05). Interestingly, we found that *Verrucomicrobia*, a unique dominant bacterium in the intestine, showed a significantly higher presence in the SS group compared to the CK group (*p* < 0.05), while *Spirochaetes*, another unique dominant bacterium found in rumen samples from both groups, displayed a significantly lower prevalence in the SS group compared to the CK group (*p* < 0.05).

#### 3.6.4 Genus level analysis

**FIGURE 9:**
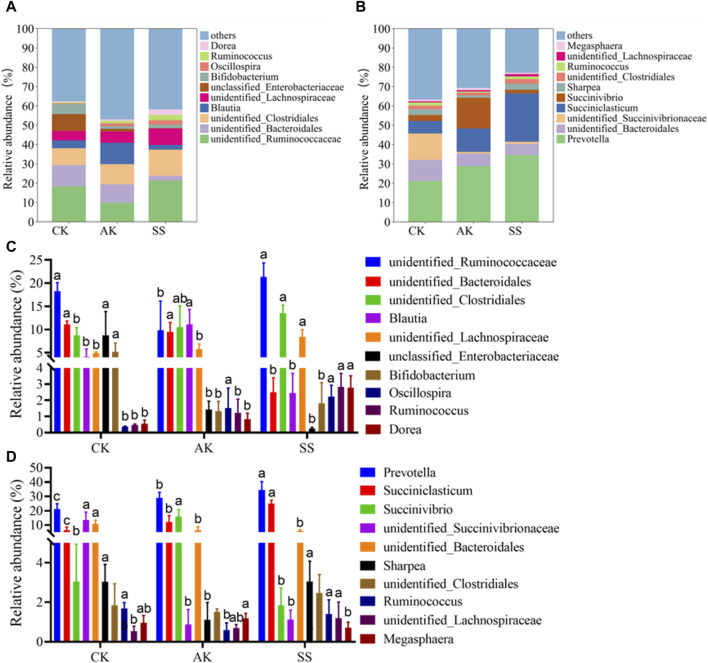
Relative abundance of the predominant microorganism at the genus level. **(A)** Predominant microorganism at the genus level in intestines, **(B)** Predominant microorganism at the genus level in rumen, **(C)** Bar chart illustrating species composition of intestinal microorganisms at the genus level, **(D)** Bar chart illustrating species composition of rumen microorganisms at the genus level. Data is presented as mean ± standard deviation (n = 6). Bars with different superscripts **(a–c)** indicate significant differences (*p* < 0.05).

As shown in Figure 9A, B, *unidentified_Clostridiales*, *unidentified_Lachnospiraceae* and *Ruminococcus* were detected in both intestinal tract and rumen of sheep lambs at the genus level. As depicted in Figure 9C, D, the intestinal SS group displayed a notably elevated relative abundance of the *unidentified_Lachnospiraceae* genus compared to both the AK group and CK group (*p* < 0.05). Similarly, in the rumen, this relative abundance was significantly higher than that in the CK group (*p* < 0.05), but no significant difference was observed when compared to the AK group (*p* > 0.05). Similarly, there was a substantial increase in the relative abundance of the *Ruminococcus* genus in the intestinal SS group compared to both AK and CK groups (*p* < 0.05). Moreover, it exhibited a significantly higher level than that in the AK group within rumen samples (*p* < 0.05), while no significant difference was found with respect to the CK group (*p* > 0.05). As shown in Figure 9C, the relative abundance of the *unidentified_Ruminococcaceae*, *unidentified_Clostridiales*, *unidentified_Lachnospiraceae*, *Oscillospira*, *Ruminococcus* and *Dorea* in the intestinal SS group were significantly higher than that in the intestinal CK group (*p* < 0.05). The relative abundance of the *unclassified Enterobacteriaceae* and *Bifidobacterium* in the intestinal SS group and AK group were significantly lower than that in the intestinal CK group (*p* < 0.05). As depicted in Figure 9D, the rumen AK group and rumen SS group exhibited a significant increase in the relative abundance of *Prevotella* and *Succiniclasticum* compared to the rumen CK group (*p* < 0.05). Conversely, there was a notable decrease (*p* < 0.05) in the relative abundance of *unidentified_Bacteroidales*.

#### 3.6.5 Metabolic function prediction analysis

**FIGURE 10:**
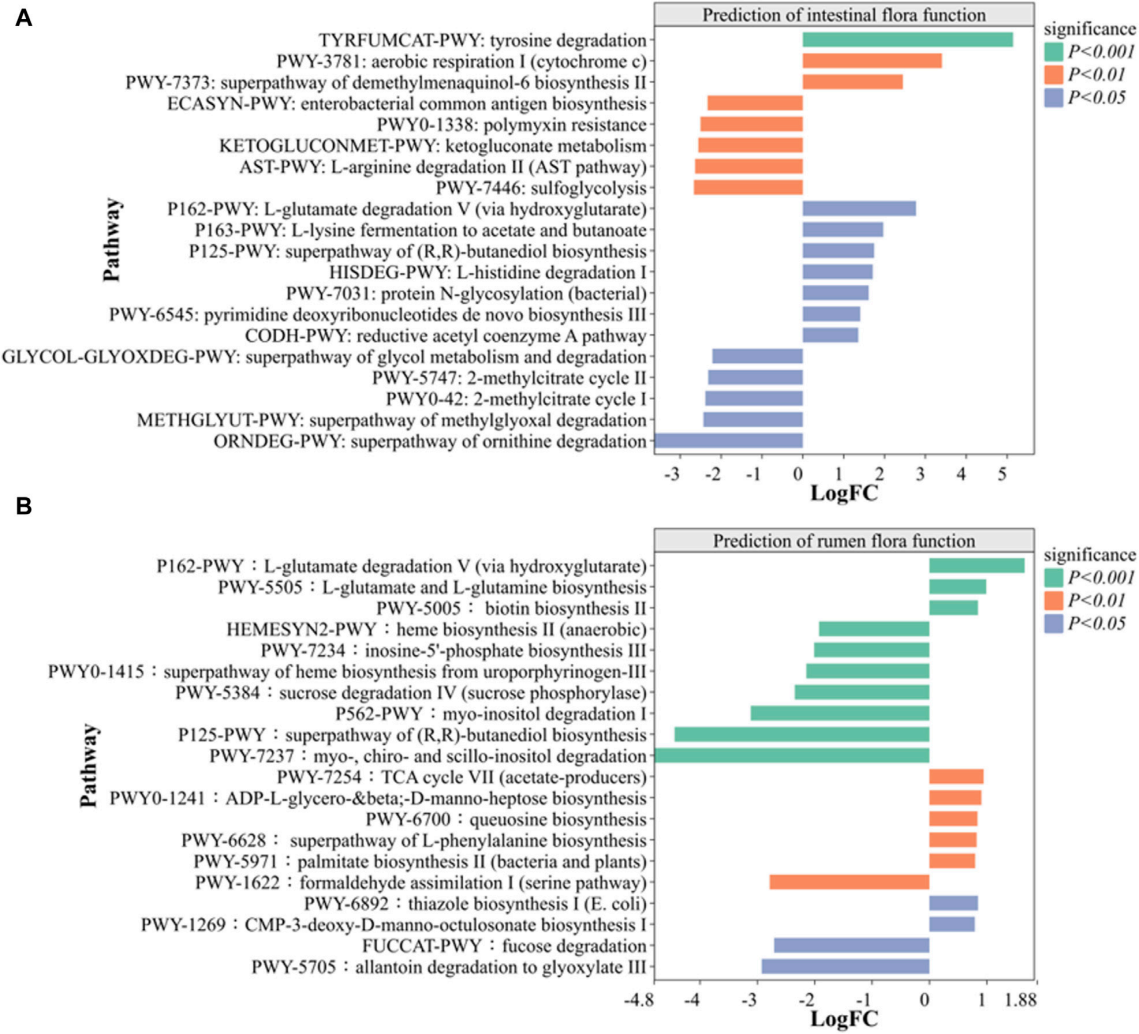
Prediction of the effect of SS on **(A)** intestinal and **(B)** rumen microorganism function in lamb based on MetaCyc pathway. (n = 6). Note: *p* < 0.05 indicate significant difference, *p* < 0.01 indicate very significant difference.

Metabolic function prediction analysis was used to identify significantly expressed metabolic pathways. A comparison with intestinal and rumen CK groups revealed 10 metabolic pathways that were significantly increased and 10 metabolic pathways that were significantly decreased in SS group (Figure 10). As shown in Figure 10A, the intestinal SS group showed a significant enhancement in the L-tyrosine degradation pathway (*p* < 0.001) and significant decrease in the superpathway of methylglyoxal degradation (*p* < 0.05) compared to the CK group. Similarly, Figure 10B demonstrates that the rumen SS group exhibited a significant enhancement in the biosynthesis pathway of L-glutamate and L-glutamine (*p* < 0.001), alongside a notable decrease in the degradation pathway of myo-, chiro- and scillo-inositol compared to the CK group. In both the intestinal and ruminal regions of sheep lambs, the SS group demonstrated a significant increase in the pathway of L-glutamate degradation V (via hydroxyglutarate) when compared to the CK group, with *p* values recorded as *p* < 0.05 and *p* < 0.01 respectively.

## 4 Discussion

Given the worldwide prohibition of antibiotics as feed additives (Castanon, 2007), there is a growing interest in exploring natural alternatives to improve animal health and optimize production outcomes (Ma et al., 2019). Plant-derived polysaccharides have emerged as promising alternatives for antibiotic-feed additives in ruminants due to their good safety, growth-promoting activity and immunomodulatory effects (Wang B. et al., 2019). Research have shown that administration of polysaccharides from Ruoqiang jujubes can enhance lamb growth and bone development by improving daily weight gain, feed intake, feed efficiency, body height, length, chest circumference and tube circumference (Moheteer et al., 2024). Table 1 shows that both the SS and AK groups had significantly greater final pipe circumference length compared to the CK group (*p* < 0.05). Additionally, the SS group exhibited significantly higher final body weight, average daily gain, and final chest length than the CK group (*p* < 0.05). These results indicated that AP from both Shanshan and Aksu could promote the growth performance of sheep lambs, with SS showing strongest effect on the growth of sheep lambs. Growth hormone (GH) is a peptide hormone released by the pituitary gland, serving a crucial function in both growth and metabolic processes (Sperling, 2016). Insulin-like growth factors-1(IGF-1) can stimulate cell growth, proliferation and differentiation, playing a significant role in animal growth (Denley et al., 2005; Wang et al., 2009). INS promotes glucose decomposition and fat synthesis (Denley et al., 2005). According to Figure 2, the levels of growth hormone (GH) and insulin (INS) in both SS and AK groups were significantly elevated compared to those in the CK group (*p* < 0.05). Furthermore, it was observed that the concentration levels in the SS group were significantly elevated compared to those in the AK group (*p* < 0.05). These results suggest that among AP collected in different regions, SS was the most effective in increasing growth hormone and insulin secretion and improving growth performance of sheep lambs.

The gastrointestinal system not only serves as the primary organ for digestion and absorption but also functioning as a crucial immune organ defending against pathogenic microorganisms (Ying et al., 2020; Cai et al., 2022b). Within the gut, the intestinal mucosal immune barrier plays a pivotal role in maintaining diverse physiological functions (Burgueño and Abreu, 2020; Wu et al., 2022), consisting of mechanical, immune, biochemical, and biological components (Burgueño and Abreu, 2020). IgA serves as the first line of defense antibody on the mucosal surface of the gastrointestinal tract, protecting the delicate intestinal lining by neutralizing potential pathogens (Bohländer et al., 2021). IgM, found in the serum and body fluids of young animals, enhances the ability of immune cells to engulf pathogenic microorganisms and combat infections (Bohländer et al., 2021). According to Figure 3, there was a significant increase in the levels of sIgA and IgM observed in both the SS group and AK group compared to the CK group (*p* < 0.05). Furthermore, it was found that the sIgA content in the SS group exhibited a notably higher level than that in the AK group (*p* < 0.05). These findings suggest that both the SS and AK groups effectively enhance intestinal sIgA secretion as well as serum IgM production, thereby boosting immune function within the intestines and throughout the body. Various cytokines such as IL-4, IL-10, IL-17, TNF-α, and INF-γ play roles in regulating IgA and IgM synthesis (Tao et al., 2014). In addition, the mucosal cytokines play a vital role in regulating the immune system of the intestines and its defense against invading pathogens (Xie et al., 2019). As shown in Figure 4, both the SS and AK groups exhibited a significant increase in the levels of IFN-γ and TNF-α compared to the CK group (*p* < 0.05). Furthermore, the SS group exhibited notably higher levels of IL-4, IL-17, and TNF-α compared to both AK and CK groups (*p* < 0.05). These findings indicate that among AP collected in different regions SS has the capability to modulate various cytokines associated with different subsets of T-helper cells (e.g., TNF-α for Th0 cells, INF-γ for Th1 cells, IL-4 for Th2 cells, IL-17 for Th17 cells), as well as Treg-associated cytokines like IL-10 (Kurashima and Kiyono, 2017). This modulation helps maintain intestinal mucosal immune homeostasis by enhancing the body’s resistance against pathogenic microorganisms and promoting immune protection within lamb intestines.

Rumen and small intestine of ruminants are the primary sites for digestion, absorption and storage of nutrients (Li et al., 2023a). As shown in Figure 5A, there were no observable physiological or structural abnormalities in the small intestine and rumen of CK, AK and SS groups, indicating that AP isolated from different regions had no adverse effects on the gastrointestinal tract of sheep lambs. Rumen papillae length and width, as well as villus height and crypt depth in small intestine are the key indicators to measure rumen and small intestine development. The increase in the length and width of the rumen papillae can enhance the absorption of nutrients in the rumen, promoting healthy rumen development (Li et al., 2023b). As shown in Figure 5A, rumen papillary height in SS group was significantly higher than that in AK and CK groups (*p* < 0.05), while rumen papillary width was intermediate between the AK and CK groups, with no significant difference observed between AK and CK groups (*p* > 0.05). The rumen papillary width in AK group was significantly higher than that in CK group (*p* < 0.05). Intestinal villi primarily serve for absorption, and increased villus expands the area available for nutrient absorption, directly influencing the growth and development of animals (Shaikh et al., 2021). The small intestine crypt, a tubular gland formed by the epithelial cells at the base of the villi invading the lamina propria, reduces in depth with enhanced cellular maturity and secretory function (Frederick et al., 2018). As shown in Figures 5B, C, the villous length of duodenum and jejunum in SS group and AK group was significantly higher than that in CK group (*p* < 0.05), while the crypt depth of duodenum and jejunum in SS group and AK group was significantly lower than that in CK group (*p* < 0.05). These findings indicate that both SS and AK have the potential to enhance digestion and absorption, promote growth, and enhance gastrointestinal immune defense by enhancing rumen papillae length, width, villi length, and crypt depth, with SS showing the most pronounced effect in all groups.

The gut microbiota plays a crucial role in maintaining the physiological and immune functions of the gastrointestinal tract, as well as promoting its health and improving lamb growth performance (Li et al., 2023a). Research indicates that incorporating herbal polysaccharides into lamb diets can enhance the abundance of microbial communities, strengthen intestinal immunity, and enhance the integrity of the intestinal barrier (Chen et al., 2021a). Among AP collected in different regions, SS showed the best activity in promoting growth, enhancing growth hormone, antibody and cytokine secretion, and improving intestinal nutrient absorption in sheep lambs. Therefore, we speculate whether SS can also increase the diversity of gastrointestinal flora and promote the number of beneficial bacteria in sheep lambs compared with AK. Thus, we utilized 16 S rRNA sequencing technology to investigate the influence of SS on the microbial composition in lamb’s rumen and intestines. In Figure 6C, it is evident that the SS group exhibited significantly higher levels of intestinal flora indices, including Chao1, Simpson, Shannon, Pielou’s evenness, Observed_species and Faith’s indices when compared to both AK and CK groups (*p* < 0.05). As shown in Figure 6D, rumen Chao1 and Observed_species indices of AK group were significantly higher than those of SS group (*p* < 0.05), while there were no significant differences in other α-diversity indices among all rumen groups (*p* > 0.05). In the evaluation of α-diversity, Chao1, Pielou’s evenness, Observed_species, and Faith_pd are utilized for quantifying the overall species count in sequencing samples. Additionally, Shannon and Simpson indices are employed to assess the richness of species diversity within the sample (Zhang et al., 2023). These findings suggest that the utilization of SS led to a notable enhancement in both the variety and abundance of the lamb’s gut microbiota. In the analysis of β-diversity, we used a Venn diagram to determine the proportion of shared and distinct microflora across various groups. Additionally, PCoA and UPGMA cluster analysis helped visualize the differentiation and clustering pattern of microflora within these groups (Zimin et al., 2021). As depicted in Figures 7A, B, the number of distinct species observed in CK, AK, and SS groups was 1741, 2,805, and 1,115 respectively. Notably, there were substantial differences in clustering patterns among the CK, AK, and SS groups (Figures 7C–F), indicating variations in intestinal flora diversity among these groups. Moreover, it can be inferred that AK and SS had the potential to enhance microbial diversity within the intestines.

To conduct a more in-depth examination of the impact of SS and AK on the composition of intestinal flora in sheep lambs, an analysis was conducted to assess the relative abundance and composition of intestinal flora at both phyla and genus levels. *Bacteroidetes* can facilitate the host to degrade various complex polysaccharides to improve nutrient absorption. It is reported that *Bacteroidetes* can slow down the inflammation of mice by promoting the secretion of IgA and IL-10 and the differentiation of Treg cells. *Firmicutes* can decompose polysaccharides and produce short-chain fatty acids such as propionic acid, butyric acid and acetic acid (Cockburn and Koropatkin, 2016). *Tenericutes* have been reported to promote gastrointestinal health and inhibit intestinal inflammation (Hu et al., 2022a). On the other hand, *Proteobacteria* and *Actinobacteria* harbor many pathogenic microorganisms. The increased abundance of *Proteobacteria* and *Actinobacteria* may disturb bacterial homeostasis and increase the risk of pathogen infection (Chen et al., 2021b). According to Figure 8, the SS group exhibited a significantly higher relative abundance of *Tenericute* in the intestine and *Firmicutes* and *Tenericutes* in the rumen compared to both AK and CK groups (*p* < 0.05). Additionally, the SS group showed a significantly higher relative abundance of *Firmicutes* in the intestine and *Verrucomicrobia* in the rumen compared to the CK group (*p* < 0.05). Moreover, there was a significant decrease in the relative abundance of *Proteobacteria* and *Actinobacteria* in the SS group compared to the CK group (*p* < 0.05). These findings indicate that the SS group has a significant impact on promoting the abundance and diversity of beneficial bacteria, while simultaneously reducing the levels of harmful *Proteobacteria* and *Actinobacteria*. Consequently, SS positively contributes to maintaining intestinal flora health when compared to both AK and CK groups. At the genus level, the relative abundance of *unidentified_Ruminococcaceae*, *unidentified_Clostridiales*, *unidentified_Lachnospiraceae*, *Oscillospira*, *Ruminococcus* and *Dorea* in SS group was significantly higher than that in CK group (*p* < 0.05) (Figures 9A, C). The relative abundance of *unclassified Enterobacteriaceae* and *Bifidobacterium* in SS and AK groups was significantly lower compared to the CK group (*p* < 0.05). In Figures 9A, C, it can be observed that the abundance of *Prevotella* and *Succiniclasticum* in SS and AK group was significantly higher than that in CK group (*p* < 0.05), while the relative abundance *unidentified_Bacteroidales* was significantly lower than that in CK group (*p* < 0.05). Studies have shown that *Lachnospiraceae*, *Ruminococcaceae*, *Oscillospira* and *Blautia* bacterial genera can decompose dietary polysaccharides and produce short-chain fatty acids through metabolism. These short-chain fatty acids provide energy for intestinal epithelial cells to repair intestinal epithelial barrier and prevent the onset of enteritis and ulcers (Benítez-Páez et al., 2020; Hu et al., 2022b). *Ruminococcus*, *Dorea* and *Prevotella* can ferment dietary fiber and promote gastrointestinal digestion and absorption. *Bifidobacterium* plays a role in maintaining gastrointestinal homeostasis, however, when the intestinal tract is compromised, intestinal *Bifidobacterium* can become pathogenic bacteria and aggravates the disease (Kuipers et al., 2024). These results indicate that compare to CK and AK, SS can enhance gastrointestinal digestion, absorption, immunity and barrier function by increasing the abundance of beneficial bacteria, while simultaneously decreasing the occurrence of gastrointestinal diseases such as enteritis and ulcers through the suppression of harmful bacteria. While the current study requires further investigation into how SS regulates the gastrointestinal flora of lambs, thereby promoting gastrointestinal function and reducing gastrointestinal diseases.

Gastrointestinal microorganisms primarily exert their influence by producing secondary metabolites through different metabolic pathways. In order to further explore the effects of SS group on the intestinal tract and rumen of sheep lambs, the MetaCyc analysis was used to compare the functional difference between SS and CK groups. As shown in Figure 10A, the intestinal SS group significantly enhanced the L-tyrosine degradation pathway (*p* < 0.001) and L-glutamate degradation V (via hydroxyglutarate) pathway (*p* < 0.05), while significantly decreasing the methylglyoxal degradation pathway (*p* < 0.05) compared to the CK group. Similarly, in Figure 10B, the rumen SS group exhibited significantly enhancement in L-glutamate and L-glutamine biosynthesis (*p* < 0.001) and L-glutamate degradation pathway (*p* < 0.01), while also decreasing myo-, chiro- and scillo-inositol degradation pathway compared to the CK group. It is reported that L-tyrosine degradation pathway and L-glutamate degradation V (via hydroxyglutarate) pathway plays an important role in the energy provision for animal cell growth and development, as well as in maintaining normal immune cell function (Hildebrandt et al., 2015). Additionally, research have shown that poultry can enhance the utilization of phytic acid and phytate utilization through the myo-inositol pathway, leading to improved feed utilization, accelerate body growth, and promoted liver fat metabolism and protection (Walk et al., 2018). Conversely, processing animal proteins or lipids through super pathway of methylglyoxal degradation promotes non-enzymatic glycosylation reactions, resulting in the production of glycosylation end products, which can have adverse effect on cells and contribute to tissue dysfunction (Phillips and Thornalley, 1993). According to the prediction of MetaCyc analysis, SS has the potential to improve gastrointestinal feed utilization, immune function, liver protection, body growth, and tissue health through comprehensive regulation of various metabolic pathways, and validation of specific metabolic pathways of SS requires further specific investigation in future studies.

## 5 Conclusion

In conclusion, among SS and AK, SS can more effectively increase the body weight and growth of sheep lambs. It also enhances the secretion of growth factor GH and INS in serum, as well as serum IgM antibody, intestinal IgA antibody and a variety of cytokines. Additionally, SS effectively promotes the growth of gastrointestinal tract, increases the type and abundance of beneficial bacteria in gastrointestinal tract, while reduce the harmful bacteria. Moreover, SS regulates a variety of immune and growth-related metabolic pathways. The SS has better effects on promoting growth and regulating intestinal flora in sheep lambs than AK, and the SS could potentially serve as an effective natural feed additive for promoting growth and regulating intestinal flora.

## Data Availability

The original contributions presented in the study are publicly available. This data can be at the NCBI platform (SRA), accession number PRJNA1104763. This is available at the following link: https://www.ncbi.nlm.nih.gov/sra/PRJNA1104763.

## Abbreviations

IL, interleukin; TNF, tumor necrosis factor; IFN, Interferon; ELISA, enzyme linked immunosorbent assay.
