# Supplementary material for: Effects of Alhagi camelorum Fisch polysaccharide from different regions on growth performance and gastrointestinal microbiota of sheep lambs
Source: Front Pharmacol. 2024 Apr 30;15:1379394. doi: 10.3389/fphar.2024.1379394 (PMC11091474; doi:10.3389/fphar.2024.1379394)
Supplement: Supplementary file 5 [file Table1.DOC]

growth performance and body size of lambs


ONEWAY FBW FBH FBL FPCL ADG BY VAR00005
  /STATISTICS DESCRIPTIVES
  /MISSING ANALYSIS
  /POSTHOC=DUNCAN LSD ALPHA(0.05).


单¦V


备ª`	
¤w创«Ø输¥X	02-FEB-2024 11:51:25	
ª`释		
输¤J	¬¡动数Õu¶°	数Õu¶°0	
	过滤¾¹	<无>	
	权­«	<无>	
	©î¤À¤å¥ó	<无>	
	¤u§@数Õu¤å¥ó¤¤ªº¦æ数	18	
¯Ê¥¢­È处²z	对¯Ê¥¢ªº©w义	将¥Î户©w义ªº¯Ê¥¢­È视为¯Ê¥¢¡C	
	¨Ï¥Îªº个®×数	¨C项¤ÀªRªº统计³£°ò¤_¨º¨Ç对¤_该¤ÀªR¤¤¨Ï¥Îªº¥ô¦ó变¶q³£¤£¨ã¦³¯Ê¥¢数Õuªº个®×¡C	
语ªk	ONEWAY FBW FBH FBL FPCL ADG BY VAR00005
  /STATISTICS DESCRIPTIVES
  /MISSING ANALYSIS
  /POSTHOC=DUNCAN LSD ALPHA(0.05).	
资·½	处²zµ{§Ç时间	00:00:00.00	
	¯Ó¥Î时间	00:00:00.03	


´y­z	
	个®×数	¥­§¡­È	标­ã®t	标­ã误®t	¥­§¡­Èªº 95% ¸m«H区间	³Ì¤p­È		
					¤U­­	¤W­­			
FBW	1.00	6	4.27	.750	.306	3.48	5.05	3		
	2.00	6	4.73	.821	.335	3.87	5.60	3		
	3.00	6	5.43	.887	.362	4.50	6.36	4		
	总计	18	4.81	.916	.216	4.36	5.27	3		
FBH	1.00	6	38.17	2.041	.833	36.02	40.31	35		
	2.00	6	41.17	1.329	.543	39.77	42.56	39		
	3.00	6	43.17	1.835	.749	41.24	45.09	40		
	总计	18	40.83	2.684	.633	39.50	42.17	35		
FBL	1.00	6	35.33	2.733	1.116	32.47	38.20	30		
	2.00	6	37.08	4.224	1.724	32.65	41.52	30		
	3.00	6	40.95	4.058	1.657	36.69	45.21	36		
	总计	18	37.79	4.257	1.003	35.67	39.91	30		
FPCL	1.00	6	5.17	.234	.095	4.92	5.41	5		
	2.00	6	5.63	.197	.080	5.43	5.84	5		
	3.00	6	6.00	.456	.186	5.52	6.48	5		
	总计	18	5.60	.460	.108	5.37	5.83	5		
ADG	1.00	6	55.00	25.966	10.601	27.75	82.25	20		
	2.00	6	74.29	24.343	9.938	48.74	99.83	27		
	3.00	6	97.62	22.607	9.229	73.89	121.34	66		
	总计	18	75.63	29.059	6.849	61.18	90.09	20		


ANOVA	
	¥­¤è©M	¦Û¥Ñ«×	§¡¤è	F	显µÛ©Ê	
FBW	组间	4.138	2	2.069	3.067	.076	
	组内	10.120	15	.675			
	总计	14.258	17				
FBH	组间	76.000	2	38.000	12.258	.001	
	组内	46.500	15	3.100			
	总计	122.500	17				
FBL	组间	99.121	2	49.561	3.559	.054	
	组内	208.897	15	13.926			
	总计	308.018	17				
FPCL	组间	2.093	2	1.047	10.420	.001	
	组内	1.507	15	.100			
	总计	3.600	17				
ADG	组间	5465.533	2	2732.766	4.611	.027	
	组内	8889.456	15	592.630			
	总计	14354.989	17				


¨Æ¦Z检验


¦h­«¤ñ较	
¦]变¶q	(I) VAR00005	(J) VAR00005	¥­§¡­È®t­È (I-J)	标­ã误®t	显µÛ©Ê	95% ¸m«H区间		
						¤U­­		
FBW	LSD	1.00	2.00	-.467	.474	.341	-1.48		
			3.00	-1.167*	.474	.027	-2.18		
		2.00	1.00	.467	.474	.341	-.54		
			3.00	-.700	.474	.161	-1.71		
		3.00	1.00	1.167*	.474	.027	.16		
			2.00	.700	.474	.161	-.31		
FBH	LSD	1.00	2.00	-3.000*	1.017	.010	-5.17		
			3.00	-5.000*	1.017	.000	-7.17		
		2.00	1.00	3.000*	1.017	.010	.83		
			3.00	-2.000	1.017	.068	-4.17		
		3.00	1.00	5.000*	1.017	.000	2.83		
			2.00	2.000	1.017	.068	-.17		
FBL	LSD	1.00	2.00	-1.750	2.155	.429	-6.34		
			3.00	-5.617*	2.155	.020	-10.21		
		2.00	1.00	1.750	2.155	.429	-2.84		
			3.00	-3.867	2.155	.093	-8.46		
		3.00	1.00	5.617*	2.155	.020	1.02		
			2.00	3.867	2.155	.093	-.73		
FPCL	LSD	1.00	2.00	-.467*	.183	.022	-.86		
			3.00	-.833*	.183	.000	-1.22		
		2.00	1.00	.467*	.183	.022	.08		
			3.00	-.367	.183	.063	-.76		
		3.00	1.00	.833*	.183	.000	.44		
			2.00	.367	.183	.063	-.02		
ADG	LSD	1.00	2.00	-19.286	14.055	.190	-49.24		
			3.00	-42.619*	14.055	.008	-72.58		
		2.00	1.00	19.286	14.055	.190	-10.67		
			3.00	-23.333	14.055	.118	-53.29		
		3.00	1.00	42.619*	14.055	.008	12.66		
			2.00	23.333	14.055	.118	-6.62		


齐©Ê¤l¶°


FBW	
	VAR00005	个®×数	Alpha ªº¤l¶° = 0.05	
			1	2	
邓ªÖa	1.00	6	4.27		
	2.00	6	4.73	4.73	
	3.00	6		5.43	
	显µÛ©Ê		.341	.161	

将显¥Ü齐©Ê¤l¶°¤¤¦U个组ªº¥­§¡­È¡C	
a. ¨Ï¥Î调©M¥­§¡­È样¥»¤j¤p = 6.000¡C	


FBH	
	VAR00005	个®×数	Alpha ªº¤l¶° = 0.05	
			1	2	
邓ªÖa	1.00	6	38.17		
	2.00	6		41.17	
	3.00	6		43.17	
	显µÛ©Ê		1.000	.068	

将显¥Ü齐©Ê¤l¶°¤¤¦U个组ªº¥­§¡­È¡C	
a. ¨Ï¥Î调©M¥­§¡­È样¥»¤j¤p = 6.000¡C	


FBL	
	VAR00005	个®×数	Alpha ªº¤l¶° = 0.05	
			1	2	
邓ªÖa	1.00	6	35.33		
	2.00	6	37.08	37.08	
	3.00	6		40.95	
	显µÛ©Ê		.429	.093	

将显¥Ü齐©Ê¤l¶°¤¤¦U个组ªº¥­§¡­È¡C	
a. ¨Ï¥Î调©M¥­§¡­È样¥»¤j¤p = 6.000¡C	


FPCL	
	VAR00005	个®×数	Alpha ªº¤l¶° = 0.05	
			1	2	
邓ªÖa	1.00	6	5.17		
	2.00	6		5.63	
	3.00	6		6.00	
	显µÛ©Ê		1.000	.063	

将显¥Ü齐©Ê¤l¶°¤¤¦U个组ªº¥­§¡­È¡C	
a. ¨Ï¥Î调©M¥­§¡­È样¥»¤j¤p = 6.000¡C	


ADG	
	VAR00005	个®×数	Alpha ªº¤l¶° = 0.05	
			1	2	
邓ªÖa	1.00	6	55.00		
	2.00	6	74.29	74.29	
	3.00	6		97.62	
	显µÛ©Ê		.190	.118	

将显¥Ü齐©Ê¤l¶°¤¤¦U个组ªº¥­§¡­È¡C	
a. ¨Ï¥Î调©M¥­§¡­È样¥»¤j¤p = 6.000¡C	


IgM
ONEWAY IgM BY VAR00001
  /STATISTICS DESCRIPTIVES
  /MISSING ANALYSIS
  /POSTHOC=DUNCAN LSD ALPHA(0.05).


单¦V

备ª`	
¤w创«Ø输¥X	02-FEB-2024 12:37:27	
ª`释		
输¤J	¬¡动数Õu¶°	数Õu¶°0	
	过滤¾¹	<无>	
	权­«	<无>	
	©î¤À¤å¥ó	<无>	
	¤u§@数Õu¤å¥ó¤¤ªº¦æ数	15	
¯Ê¥¢­È处²z	对¯Ê¥¢ªº©w义	将¥Î户©w义ªº¯Ê¥¢­È视为¯Ê¥¢¡C	
	¨Ï¥Îªº个®×数	¨C项¤ÀªRªº统计³£°ò¤_¨º¨Ç对¤_该¤ÀªR¤¤¨Ï¥Îªº¥ô¦ó变¶q³£¤£¨ã¦³¯Ê¥¢数Õuªº个®×¡C	
语ªk	ONEWAY IgM BY VAR00001
  /STATISTICS DESCRIPTIVES
  /MISSING ANALYSIS
  /POSTHOC=DUNCAN LSD ALPHA(0.05).	
资·½	处²zµ{§Ç时间	00:00:00.00	
	¯Ó¥Î时间	00:00:00.01	


[数Õu¶°0] 

´y­z	
IgM  	
	个®×数	¥­§¡­È	标­ã®t	标­ã误®t	¥­§¡­Èªº 95% ¸m«H区间	³Ì¤p­È	³Ì¤j­È	
					¤U­­	¤W­­			
1.00	5	6.8410	.68259	.30526	5.9934	7.6885	5.81	7.53	
2.00	5	7.2851	.60061	.26860	6.5393	8.0309	6.67	8.03	
3.00	5	8.2020	.60698	.27145	7.4483	8.9557	7.24	8.89	
总计	15	7.4427	.82798	.21378	6.9842	7.9012	5.81	8.89	


ANOVA	
IgM  	
	¥­¤è©M	¦Û¥Ñ«×	§¡¤è	F	显µÛ©Ê	
组间	4.817	2	2.409	6.046	.015	
组内	4.780	12	.398			
总计	9.598	14				


¨Æ¦Z检验


¦h­«¤ñ较	
¦]变¶q:   IgM  	
	(I) VAR00001	(J) VAR00001	¥­§¡­È®t­È (I-J)	标­ã误®t	显µÛ©Ê	95% ¸m«H区间		
						¤U­­		
LSD	1.00	2.00	-.44413	.39918	.288	-1.3139		
		3.00	-1.36103*	.39918	.005	-2.2308		
	2.00	1.00	.44413	.39918	.288	-.4256		
		3.00	-.91691*	.39918	.040	-1.7866		
	3.00	1.00	1.36103*	.39918	.005	.4913		
		2.00	.91691*	.39918	.040	.0472		

齐©Ê¤l¶°

IgM	
	VAR00001	个®×数	Alpha ªº¤l¶° = 0.05	
			1	2	
邓ªÖa	1.00	5	6.8410		
	2.00	5	7.2851		
	3.00	5		8.2020	
	显µÛ©Ê		.288	1.000	

将显¥Ü齐©Ê¤l¶°¤¤¦U个组ªº¥­§¡­È¡C	
a. ¨Ï¥Î调©M¥­§¡­È样¥»¤j¤p = 5.000¡C	


Intestinal and rumen morphological data
ONEWAY DVH DCd JVH JCd RnH RpW BY VAR00001
  /STATISTICS DESCRIPTIVES
  /MISSING ANALYSIS
  /POSTHOC=DUNCAN LSD ALPHA(0.05).


单¦V


备ª`	
¤w创«Ø输¥X	02-FEB-2024 13:30:07	
ª`释		
输¤J	¬¡动数Õu¶°	数Õu¶°0	
	过滤¾¹	<无>	
	权­«	<无>	
	©î¤À¤å¥ó	<无>	
	¤u§@数Õu¤å¥ó¤¤ªº¦æ数	15	
¯Ê¥¢­È处²z	对¯Ê¥¢ªº©w义	将¥Î户©w义ªº¯Ê¥¢­È视为¯Ê¥¢¡C	
	¨Ï¥Îªº个®×数	¨C项¤ÀªRªº统计³£°ò¤_¨º¨Ç对¤_该¤ÀªR¤¤¨Ï¥Îªº¥ô¦ó变¶q³£¤£¨ã¦³¯Ê¥¢数Õuªº个®×¡C	
语ªk	ONEWAY DVH DCd JVH JCd RnH RpW BY VAR00001
  /STATISTICS DESCRIPTIVES
  /MISSING ANALYSIS
  /POSTHOC=DUNCAN LSD ALPHA(0.05).	
资·½	处²zµ{§Ç时间	00:00:00.00	
	¯Ó¥Î时间	00:00:00.03	


[数Õu¶°0] 

´y­z	
	个®×数	¥­§¡­È	标­ã®t	标­ã误®t	¥­§¡­Èªº 95% ¸m«H区间	³Ì¤p­È		
					¤U­­	¤W­­			
DVH	1.00	5	607.82	75.240	33.649	514.40	701.25	498		
	2.00	5	759.67	44.528	19.914	704.38	814.96	705		
	3.00	5	800.42	44.528	19.913	745.13	855.71	736		
	总计	15	722.64	100.544	25.960	666.96	778.32	498		
DCd	1.00	5	270.11	30.384	13.588	232.38	307.83	236		
	2.00	5	209.81	17.059	7.629	188.63	230.99	188		
	3.00	5	189.52	15.335	6.858	170.48	208.56	168		
	总计	15	223.15	40.855	10.549	200.52	245.77	168		
JVH	1.00	5	601.48	27.495	12.296	567.34	635.62	560		
	2.00	5	760.67	39.904	17.846	711.12	810.22	719		
	3.00	5	765.47	53.147	23.768	699.48	831.46	687		
	总计	15	709.21	87.744	22.655	660.62	757.80	560		
JCd	1.00	5	253.40	8.660	3.873	242.65	264.15	242		
	2.00	5	226.74	4.710	2.106	220.89	232.59	219		
	3.00	5	191.80	13.677	6.116	174.82	208.78	173		
	总计	15	223.98	27.621	7.132	208.68	239.28	173		
RnH	1.00	5	569.27	65.775	29.416	487.60	650.94	460		
	2.00	5	502.68	41.265	18.454	451.44	553.92	446		
	3.00	5	967.05	82.480	36.886	864.64	1069.46	869		
	总计	15	679.67	220.686	56.981	557.46	801.88	446		
RpW	1.00	5	293.39	30.228	13.519	255.86	330.92	258		
	2.00	5	345.01	24.945	11.156	314.03	375.98	310		
	3.00	5	316.76	21.864	9.778	289.61	343.91	294		
	总计	15	318.39	32.444	8.377	300.42	336.35	258		


ANOVA	
	¥­¤è©M	¦Û¥Ñ«×	§¡¤è	F	显µÛ©Ê	
DVH	组间	103021.353	2	51510.676	16.053	.000	
	组内	38506.280	12	3208.857			
	总计	141527.633	14				
DCd	组间	17569.931	2	8784.966	18.184	.000	
	组内	5797.431	12	483.119			
	总计	23367.362	14				
JVH	组间	87093.302	2	43546.651	25.254	.000	
	组内	20691.897	12	1724.325			
	总计	107785.199	14				
JCd	组间	9543.642	2	4771.821	50.366	.000	
	组内	1136.919	12	94.743			
	总计	10680.561	14				
RnH	组间	630504.422	2	315252.211	73.702	.000	
	组内	51328.507	12	4277.376			
	总计	681832.930	14				
RpW	组间	6680.309	2	3340.154	4.975	.027	
	组内	8056.228	12	671.352			
	总计	14736.537	14				


¨Æ¦Z检验


¦h­«¤ñ较	
¦]变¶q	(I) VAR00001	(J) VAR00001	¥­§¡­È®t­È (I-J)	标­ã误®t	显µÛ©Ê	95% ¸m«H区间	
						¤U­­	¤W­­	
DVH	LSD	1.00	2.00	-151.850*	35.827	.001	-229.91	-73.79	
			3.00	-192.598*	35.827	.000	-270.66	-114.54	
		2.00	1.00	151.850*	35.827	.001	73.79	229.91	
			3.00	-40.748	35.827	.278	-118.81	37.31	
		3.00	1.00	192.598*	35.827	.000	114.54	270.66	
			2.00	40.748	35.827	.278	-37.31	118.81	
DCd	LSD	1.00	2.00	60.298*	13.901	.001	30.01	90.59	
			3.00	80.588*	13.901	.000	50.30	110.88	
		2.00	1.00	-60.298*	13.901	.001	-90.59	-30.01	
			3.00	20.290	13.901	.170	-10.00	50.58	
		3.00	1.00	-80.588*	13.901	.000	-110.88	-50.30	
			2.00	-20.290	13.901	.170	-50.58	10.00	
JVH	LSD	1.00	2.00	-159.186*	26.263	.000	-216.41	-101.96	
			3.00	-163.990*	26.263	.000	-221.21	-106.77	
		2.00	1.00	159.186*	26.263	.000	101.96	216.41	
			3.00	-4.804	26.263	.858	-62.03	52.42	
		3.00	1.00	163.990*	26.263	.000	106.77	221.21	
			2.00	4.804	26.263	.858	-52.42	62.03	
JCd	LSD	1.00	2.00	26.656*	6.156	.001	13.24	40.07	
			3.00	61.600*	6.156	.000	48.19	75.01	
		2.00	1.00	-26.656*	6.156	.001	-40.07	-13.24	
			3.00	34.944*	6.156	.000	21.53	48.36	
		3.00	1.00	-61.600*	6.156	.000	-75.01	-48.19	
			2.00	-34.944*	6.156	.000	-48.36	-21.53	
RnH	LSD	1.00	2.00	66.590	41.364	.133	-23.53	156.71	
			3.00	-397.780*	41.364	.000	-487.90	-307.66	
		2.00	1.00	-66.590	41.364	.133	-156.71	23.53	
			3.00	-464.370*	41.364	.000	-554.49	-374.25	
		3.00	1.00	397.780*	41.364	.000	307.66	487.90	
			2.00	464.370*	41.364	.000	374.25	554.49	
RpW	LSD	1.00	2.00	-51.616*	16.387	.008	-87.32	-15.91	
			3.00	-23.372	16.387	.179	-59.08	12.33	
		2.00	1.00	51.616*	16.387	.008	15.91	87.32	
			3.00	28.244	16.387	.110	-7.46	63.95	
		3.00	1.00	23.372	16.387	.179	-12.33	59.08	
			2.00	-28.244	16.387	.110	-63.95	7.46	

*. ¥­§¡­È®t­Èªº显µÛ©Ê¤ô¥­为 0.05¡C	


齐©Ê¤l¶°


DVH	
	VAR00001	个®×数	Alpha ªº¤l¶° = 0.05	
			1	2	
邓ªÖa	1.00	5	607.82		
	2.00	5		759.67	
	3.00	5		800.42	
	显µÛ©Ê		1.000	.278	

将显¥Ü齐©Ê¤l¶°¤¤¦U个组ªº¥­§¡­È¡C	
a. ¨Ï¥Î调©M¥­§¡­È样¥»¤j¤p = 5.000¡C	

DCd	
	VAR00001	个®×数	Alpha ªº¤l¶° = 0.05	
			1	2	
邓ªÖa	3.00	5	189.52		
	2.00	5	209.81		
	1.00	5		270.11	
	显µÛ©Ê		.170	1.000	

将显¥Ü齐©Ê¤l¶°¤¤¦U个组ªº¥­§¡­È¡C	
a. ¨Ï¥Î调©M¥­§¡­È样¥»¤j¤p = 5.000¡C	

JVH	
	VAR00001	个®×数	Alpha ªº¤l¶° = 0.05	
			1	2	
邓ªÖa	1.00	5	601.48		
	2.00	5		760.67	
	3.00	5		765.47	
	显µÛ©Ê		1.000	.858	

将显¥Ü齐©Ê¤l¶°¤¤¦U个组ªº¥­§¡­È¡C	
a. ¨Ï¥Î调©M¥­§¡­È样¥»¤j¤p = 5.000¡C	

JCd	
	VAR00001	个®×数	Alpha ªº¤l¶° = 0.05	
			1	2	3	
邓ªÖa	3.00	5	191.80			
	2.00	5		226.74		
	1.00	5			253.40	
	显µÛ©Ê		1.000	1.000	1.000	

将显¥Ü齐©Ê¤l¶°¤¤¦U个组ªº¥­§¡­È¡C	
a. ¨Ï¥Î调©M¥­§¡­È样¥»¤j¤p = 5.000¡C	


RnH	
	VAR00001	个®×数	Alpha ªº¤l¶° = 0.05	
			1	2	
邓ªÖa	2.00	5	502.68		
	1.00	5	569.27		
	3.00	5		967.05	
	显µÛ©Ê		.133	1.000	

将显¥Ü齐©Ê¤l¶°¤¤¦U个组ªº¥­§¡­È¡C	
a. ¨Ï¥Î调©M¥­§¡­È样¥»¤j¤p = 5.000¡C	


RpW	
	VAR00001	个®×数	Alpha ªº¤l¶° = 0.05	
			1	2	
邓ªÖa	1.00	5	293.39		
	3.00	5	316.76	316.76	
	2.00	5		345.01	
	显µÛ©Ê		.179	.110	

将显¥Ü齐©Ê¤l¶°¤¤¦U个组ªº¥­§¡­È¡C	
a. ¨Ï¥Î调©M¥­§¡­È样¥»¤j¤p = 5.000¡C	
phylum level of rumen main microorganism


ONEWAY Bacteroidetes Firmicutes Proteobacteria Spirochaetes Actinobacteria Tenericutes BY VAR00001
  /STATISTICS DESCRIPTIVES
  /MISSING ANALYSIS
  /POSTHOC=DUNCAN LSD ALPHA(0.05).


单¦V


备ª`	
¤w创«Ø输¥X	22-DEC-2023 12:06:20	
ª`释		
输¤J	¬¡动数Õu¶°	数Õu¶°0	
	过滤¾¹	<无>	
	权­«	<无>	
	©î¤À¤å¥ó	<无>	
	¤u§@数Õu¤å¥ó¤¤ªº¦æ数	18	
¯Ê¥¢­È处²z	对¯Ê¥¢ªº©w义	将¥Î户©w义ªº¯Ê¥¢­È视为¯Ê¥¢¡C	
	¨Ï¥Îªº个®×数	¨C项¤ÀªRªº统计³£°ò¤_¨º¨Ç对¤_该¤ÀªR¤¤¨Ï¥Îªº¥ô¦ó变¶q³£¤£¨ã¦³¯Ê¥¢数Õuªº个®×¡C	
语ªk	ONEWAY Bacteroidetes Firmicutes Proteobacteria Spirochaetes Actinobacteria Tenericutes BY VAR00001
  /STATISTICS DESCRIPTIVES
  /MISSING ANALYSIS
  /POSTHOC=DUNCAN LSD ALPHA(0.05).	
资·½	处²zµ{§Ç时间	00:00:00.00	
	¯Ó¥Î时间	00:00:00.03	


[数Õu¶°0] 


´y­z	
	个®×数	¥­§¡­È	标­ã®t	标­ã误®t	¥­§¡­Èªº 95% ¸m«H区间			
					¤U­­	¤W­­			
Bacteroidetes	1.00	6	35.8857	4.38068	1.78840	31.2884	40.4829			
	2.00	6	36.1005	2.54291	1.03814	33.4319	38.7691			
	3.00	6	44.2742	7.47950	3.05349	36.4249	52.1234			
	总计	18	38.7534	6.33590	1.49339	35.6027	41.9042			
Firmicutes	1.00	6	29.2163	5.27811	2.15478	23.6773	34.7554			
	2.00	6	33.4283	1.95215	.79696	31.3797	35.4770			
	3.00	6	48.1153	5.34355	2.18150	42.5076	53.7230			
	总计	18	36.9200	9.33796	2.20098	32.2763	41.5637			
Proteobacteria	1.00	6	13.2765	6.14103	2.50706	6.8319	19.7211			
	2.00	6	18.9177	4.03152	1.64586	14.6868	23.1485			
	3.00	6	4.2973	1.63814	.66877	2.5782	6.0165			
	总计	18	12.1638	7.41877	1.74862	8.4746	15.8531			
Spirochaetes	1.00	6	16.8982	6.82211	2.78512	9.7388	24.0575			
	2.00	6	3.6422	2.18882	.89358	1.3451	5.9392			
	3.00	6	.1059	.03230	.01319	.0720	.1398			
	总计	18	6.8821	8.39145	1.97788	2.7091	11.0551			
Actinobacteria	1.00	6	.9418	.43509	.17763	.4852	1.3984			
	2.00	6	5.6590	3.47278	1.41776	2.0145	9.3035			
	3.00	6	.6742	.17605	.07187	.4894	.8589			
	总计	18	2.4250	3.02681	.71342	.9198	3.9302			
Tenericutes	1.00	6	.2718	.10600	.04328	.1606	.3831			
	2.00	6	.3945	.19938	.08140	.1853	.6037			
	3.00	6	2.0258	.74551	.30435	1.2435	2.8082			
	总计	18	.8974	.92481	.21798	.4375	1.3573			


ANOVA	
	¥­¤è©M	¦Û¥Ñ«×	§¡¤è	F	显µÛ©Ê	
Bacteroidetes	组间	274.444	2	137.222	5.045	.021	
	组内	407.998	15	27.200			
	总计	682.442	17				
Firmicutes	组间	1181.242	2	590.621	29.422	.000	
	组内	301.114	15	20.074			
	总计	1482.357	17				
Proteobacteria	组间	652.405	2	326.202	17.275	.000	
	组内	283.244	15	18.883			
	总计	935.649	17				
Spirochaetes	组间	940.413	2	470.206	27.480	.000	
	组内	256.666	15	17.111			
	总计	1197.078	17				
Actinobacteria	组间	94.344	2	47.172	11.524	.001	
	组内	61.403	15	4.094			
	总计	155.746	17				
Tenericutes	组间	11.506	2	5.753	28.443	.000	
	组内	3.034	15	.202			
	总计	14.540	17				


¨Æ¦Z检验


¦h­«¤ñ较	
¦]变¶q	(I) VAR00001	(J) VAR00001	¥­§¡­È®t­È (I-J)	标­ã误®t	显µÛ©Ê	95% ¸m«H区间	
						¤U­­	¤W­­	
Bacteroidetes	LSD	1.00	2.00	-.21483	3.01108	.944	-6.6328	6.2031	
			3.00	-8.38850*	3.01108	.014	-14.8065	-1.9705	
		2.00	1.00	.21483	3.01108	.944	-6.2031	6.6328	
			3.00	-8.17367*	3.01108	.016	-14.5916	-1.7557	
		3.00	1.00	8.38850*	3.01108	.014	1.9705	14.8065	
			2.00	8.17367*	3.01108	.016	1.7557	14.5916	
Firmicutes	LSD	1.00	2.00	-4.21200	2.58678	.124	-9.7256	1.3016	
			3.00	-18.89900*	2.58678	.000	-24.4126	-13.3854	
		2.00	1.00	4.21200	2.58678	.124	-1.3016	9.7256	
			3.00	-14.68700*	2.58678	.000	-20.2006	-9.1734	
		3.00	1.00	18.89900*	2.58678	.000	13.3854	24.4126	
			2.00	14.68700*	2.58678	.000	9.1734	20.2006	
Proteobacteria	LSD	1.00	2.00	-5.64117*	2.50885	.040	-10.9887	-.2937	
			3.00	8.97917*	2.50885	.003	3.6317	14.3267	
		2.00	1.00	5.64117*	2.50885	.040	.2937	10.9887	
			3.00	14.62033*	2.50885	.000	9.2728	19.9678	
		3.00	1.00	-8.97917*	2.50885	.003	-14.3267	-3.6317	
			2.00	-14.62033*	2.50885	.000	-19.9678	-9.2728	
Spirochaetes	LSD	1.00	2.00	13.25600*	2.38824	.000	8.1656	18.3464	
			3.00	16.79225*	2.38824	.000	11.7018	21.8827	
		2.00	1.00	-13.25600*	2.38824	.000	-18.3464	-8.1656	
			3.00	3.53625	2.38824	.159	-1.5542	8.6267	
		3.00	1.00	-16.79225*	2.38824	.000	-21.8827	-11.7018	
			2.00	-3.53625	2.38824	.159	-8.6267	1.5542	
Actinobacteria	LSD	1.00	2.00	-4.71717*	1.16812	.001	-7.2070	-2.2274	
			3.00	.26767	1.16812	.822	-2.2221	2.7575	
		2.00	1.00	4.71717*	1.16812	.001	2.2274	7.2070	
			3.00	4.98483*	1.16812	.001	2.4950	7.4746	
		3.00	1.00	-.26767	1.16812	.822	-2.7575	2.2221	
			2.00	-4.98483*	1.16812	.001	-7.4746	-2.4950	
Tenericutes	LSD	1.00	2.00	-.12267	.25965	.643	-.6761	.4308	
			3.00	-1.75400*	.25965	.000	-2.3074	-1.2006	
		2.00	1.00	.12267	.25965	.643	-.4308	.6761	
			3.00	-1.63133*	.25965	.000	-2.1848	-1.0779	
		3.00	1.00	1.75400*	.25965	.000	1.2006	2.3074	
			2.00	1.63133*	.25965	.000	1.0779	2.1848	

*. ¥­§¡­È®t­Èªº显µÛ©Ê¤ô¥­为 0.05¡C	


齐©Ê¤l¶°


Bacteroidetes	
	VAR00001	个®×数	Alpha ªº¤l¶° = 0.05	
			1	2	
邓ªÖa	1.00	6	35.8857		
	2.00	6	36.1005		
	3.00	6		44.2742	
	显µÛ©Ê		.944	1.000	

将显¥Ü齐©Ê¤l¶°¤¤¦U个组ªº¥­§¡­È¡C	
a. ¨Ï¥Î调©M¥­§¡­È样¥»¤j¤p = 6.000¡C	


Firmicutes	
	VAR00001	个®×数	Alpha ªº¤l¶° = 0.05	
			1	2	
邓ªÖa	1.00	6	29.2163		
	2.00	6	33.4283		
	3.00	6		48.1153	
	显µÛ©Ê		.124	1.000	

将显¥Ü齐©Ê¤l¶°¤¤¦U个组ªº¥­§¡­È¡C	
a. ¨Ï¥Î调©M¥­§¡­È样¥»¤j¤p = 6.000¡C	


Proteobacteria	
	VAR00001	个®×数	Alpha ªº¤l¶° = 0.05	
			1	2	3	
邓ªÖa	3.00	6	4.2973			
	1.00	6		13.2765		
	2.00	6			18.9177	
	显µÛ©Ê		1.000	1.000	1.000	

将显¥Ü齐©Ê¤l¶°¤¤¦U个组ªº¥­§¡­È¡C	
a. ¨Ï¥Î调©M¥­§¡­È样¥»¤j¤p = 6.000¡C	


Spirochaetes	
	VAR00001	个®×数	Alpha ªº¤l¶° = 0.05	
			1	2	
邓ªÖa	3.00	6	.1059		
	2.00	6	3.6422		
	1.00	6		16.8982	
	显µÛ©Ê		.159	1.000	

将显¥Ü齐©Ê¤l¶°¤¤¦U个组ªº¥­§¡­È¡C	
a. ¨Ï¥Î调©M¥­§¡­È样¥»¤j¤p = 6.000¡C	


Actinobacteria	
	VAR00001	个®×数	Alpha ªº¤l¶° = 0.05	
			1	2	
邓ªÖa	3.00	6	.6742		
	1.00	6	.9418		
	2.00	6		5.6590	
	显µÛ©Ê		.822	1.000	

将显¥Ü齐©Ê¤l¶°¤¤¦U个组ªº¥­§¡­È¡C	
a. ¨Ï¥Î调©M¥­§¡­È样¥»¤j¤p = 6.000¡C	


Tenericutes	
	VAR00001	个®×数	Alpha ªº¤l¶° = 0.05	
			1	2	
邓ªÖa	1.00	6	.2718		
	2.00	6	.3945		
	3.00	6		2.0258	
	显µÛ©Ê		.643	1.000	

将显¥Ü齐©Ê¤l¶°¤¤¦U个组ªº¥­§¡­È¡C	
a. ¨Ï¥Î调©M¥­§¡­È样¥»¤j¤p = 6.000¡C	


serum growth factorserum growth factor

ONEWAY GH IGF1 INS BY VAR00005
  /STATISTICS DESCRIPTIVES
  /MISSING ANALYSIS
  /POSTHOC=DUNCAN LSD ALPHA(0.05).


单¦V


备ª`	
¤w创«Ø输¥X	02-FEB-2024 11:16:37	
ª`释		
输¤J	¬¡动数Õu¶°	数Õu¶°0	
	过滤¾¹	<无>	
	权­«	<无>	
	©î¤À¤å¥ó	<无>	
	¤u§@数Õu¤å¥ó¤¤ªº¦æ数	15	
¯Ê¥¢­È处²z	对¯Ê¥¢ªº©w义	将¥Î户©w义ªº¯Ê¥¢­È视为¯Ê¥¢¡C	
	¨Ï¥Îªº个®×数	¨C项¤ÀªRªº统计³£°ò¤_¨º¨Ç对¤_该¤ÀªR¤¤¨Ï¥Îªº¥ô¦ó变¶q³£¤£¨ã¦³¯Ê¥¢数Õuªº个®×¡C	
语ªk	ONEWAY GH IGF1 INS BY VAR00005
  /STATISTICS DESCRIPTIVES
  /MISSING ANALYSIS
  /POSTHOC=DUNCAN LSD ALPHA(0.05).	
资·½	处²zµ{§Ç时间	00:00:00.00	
	¯Ó¥Î时间	00:00:00.02	


[数Õu¶°0] 


´y­z	
	个®×数	¥­§¡­È	标­ã®t	标­ã误®t	¥­§¡­Èªº 95% ¸m«H区间	³Ì¤p­È		
					¤U­­	¤W­­			
GH	1.00	5	4.2544	.16630	.07437	4.0479	4.4609	4.08		
	2.00	5	5.2630	.74937	.33513	4.3325	6.1935	4.63		
	3.00	5	7.5220	.77772	.34780	6.5563	8.4877	6.49		
	总计	15	5.6798	1.52999	.39504	4.8325	6.5271	4.08		
IGF1	1.00	5	448.3332	59.11675	26.43782	374.9301	521.7363	375.00		
	2.00	5	499.1668	106.28671	47.53286	367.1944	631.1392	375.00		
	3.00	5	524.9998	48.94727	21.88989	464.2237	585.7759	470.83		
	总计	15	490.8333	77.44364	19.99586	447.9464	533.7201	375.00		
INS	1.00	5	14.4444	2.13706	.95572	11.7909	17.0979	10.78		
	2.00	5	27.0000	2.09349	.93624	24.4006	29.5994	25.22		
	3.00	5	45.6667	6.80867	3.04493	37.2126	54.1208	34.67		
	总计	15	29.0370	13.86008	3.57866	21.3616	36.7125	10.78		


ANOVA	
	¥­¤è©M	¦Û¥Ñ«×	§¡¤è	F	显µÛ©Ê	
GH	组间	27.996	2	13.998	35.169	.000	
	组内	4.776	12	.398			
	总计	32.772	14				
IGF1	组间	15215.277	2	7607.639	1.328	.301	
	组内	68749.960	12	5729.163			
	总计	83965.237	14				
INS	组间	2468.196	2	1234.098	66.940	.000	
	组内	221.231	12	18.436			
	总计	2689.427	14				


¨Æ¦Z检验


¦h­«¤ñ较	
¦]变¶q	(I) VAR00005	(J) VAR00005	¥­§¡­È®t­È (I-J)	标­ã误®t	显µÛ©Ê	95% ¸m«H区间		
						¤U­­		
GH	LSD	1.00	2.00	-1.00860*	.39901	.027	-1.8780		
			3.00	-3.26760*	.39901	.000	-4.1370		
		2.00	1.00	1.00860*	.39901	.027	.1392		
			3.00	-2.25900*	.39901	.000	-3.1284		
		3.00	1.00	3.26760*	.39901	.000	2.3982		
			2.00	2.25900*	.39901	.000	1.3896		
IGF1	LSD	1.00	2.00	-50.83360	47.87134	.309	-155.1363		
			3.00	-76.66660	47.87134	.135	-180.9693		
		2.00	1.00	50.83360	47.87134	.309	-53.4691		
			3.00	-25.83300	47.87134	.599	-130.1357		
		3.00	1.00	76.66660	47.87134	.135	-27.6361		
			2.00	25.83300	47.87134	.599	-78.4697		
INS	LSD	1.00	2.00	-12.55560*	2.71558	.001	-18.4723		
			3.00	-31.22227*	2.71558	.000	-37.1390		
		2.00	1.00	12.55560*	2.71558	.001	6.6389		
			3.00	-18.66667*	2.71558	.000	-24.5834		
		3.00	1.00	31.22227*	2.71558	.000	25.3055		
			2.00	18.66667*	2.71558	.000	12.7499		


齐©Ê¤l¶°


GH	
	VAR00005	个®×数	Alpha ªº¤l¶° = 0.05	
			1	2	3	
邓ªÖa	1.00	5	4.2544			
	2.00	5		5.2630		
	3.00	5			7.5220	
	显µÛ©Ê		1.000	1.000	1.000	

将显¥Ü齐©Ê¤l¶°¤¤¦U个组ªº¥­§¡­È¡C	
a. ¨Ï¥Î调©M¥­§¡­È样¥»¤j¤p = 5.000¡C	


IGF1	
	VAR00005	个®×数	Alpha ªº¤l¶° = 0.05	
			1	
邓ªÖa	1.00	5	448.3332	
	2.00	5	499.1668	
	3.00	5	524.9998	
	显µÛ©Ê		.153	

将显¥Ü齐©Ê¤l¶°¤¤¦U个组ªº¥­§¡­È¡C	
a. ¨Ï¥Î调©M¥­§¡­È样¥»¤j¤p = 5.000¡C	


INS	
	VAR00005	个®×数	Alpha ªº¤l¶° = 0.05	
			1	2	3	
邓ªÖa	1.00	5	14.4444			
	2.00	5		27.0000		
	3.00	5			45.6667	
	显µÛ©Ê		1.000	1.000	1.000	

将显¥Ü齐©Ê¤l¶°¤¤¦U个组ªº¥­§¡­È¡C	
a. ¨Ï¥Î调©M¥­§¡­È样¥»¤j¤p = 5.000¡C	


The genus level of intestinal main microorganism


ONEWAY unidentified_Ruminococcaceae unidentified_Bacteroidales unidentified_Clostridiales Blautia
    unidentified_Lachnospiraceae unclassified_Enterobacteriaceae Bifidobacterium Oscillospira
    Ruminococcus Dorea BY VAR00001
  /STATISTICS DESCRIPTIVES
  /MISSING ANALYSIS
  /POSTHOC=DUNCAN LSD ALPHA(0.05).


单¦V


备ª`	
¤w创«Ø输¥X	10-JAN-2024 18:22:01	
ª`释		
输¤J	¬¡动数Õu¶°	数Õu¶°0	
	过滤¾¹	<无>	
	权­«	<无>	
	©î¤À¤å¥ó	<无>	
	¤u§@数Õu¤å¥ó¤¤ªº¦æ数	18	
¯Ê¥¢­È处²z	对¯Ê¥¢ªº©w义	将¥Î户©w义ªº¯Ê¥¢­È视为¯Ê¥¢¡C	
	¨Ï¥Îªº个®×数	¨C项¤ÀªRªº统计³£°ò¤_¨º¨Ç对¤_该¤ÀªR¤¤¨Ï¥Îªº¥ô¦ó变¶q³£¤£¨ã¦³¯Ê¥¢数Õuªº个®×¡C	
语ªk	ONEWAY unidentified_Ruminococcaceae unidentified_Bacteroidales unidentified_Clostridiales Blautia
    unidentified_Lachnospiraceae unclassified_Enterobacteriaceae Bifidobacterium Oscillospira
    Ruminococcus Dorea BY VAR00001
  /STATISTICS DESCRIPTIVES
  /MISSING ANALYSIS
  /POSTHOC=DUNCAN LSD ALPHA(0.05).	
资·½	处²zµ{§Ç时间	00:00:00.00	
	¯Ó¥Î时间	00:00:00.05	


[数Õu¶°0] 


´y­z	
	个®×数	¥­§¡­È	标­ã®t	标­ã误®t	¥­§¡­Èªº 95% ¸m«H区间	³Ì¤p­È		
					¤U­­	¤W­­			
unidentified_Ruminococcaceae	1	6	18.2659	1.84281	.75233	16.3320	20.1998	16.37		
	2	6	9.8652	6.26555	2.55790	3.2899	16.4404	5.60		
	3	6	21.3177	3.06472	1.25117	18.1015	24.5339	17.38		
	总计	18	16.4829	6.33535	1.49326	13.3324	19.6334	5.60		
unidentified_Bacteroidales	1	6	11.1172	.72213	.29481	10.3593	11.8750	9.91		
	2	6	9.4908	2.05186	.83767	7.3375	11.6441	8.01		
	3	6	2.5023	.88096	.35965	1.5778	3.4268	1.55		
	总计	18	7.7034	4.05070	.95476	5.6891	9.7178	1.55		
unidentified_Clostridiales	1	6	8.6904	1.69957	.69384	6.9068	10.4740	6.66		
	2	6	10.4776	4.62366	1.88760	5.6253	15.3298	6.45		
	3	6	13.5259	1.71728	.70108	11.7237	15.3280	10.77		
	总计	18	10.8979	3.49634	.82410	9.1592	12.6366	6.45		
Blautia	1	6	4.0806	1.70730	.69700	2.2889	5.8723	2.37		
	2	6	11.0731	3.26463	1.33278	7.6471	14.4992	5.49		
	3	6	2.4492	1.22090	.49843	1.1679	3.7304	.74		
	总计	18	5.8676	4.38699	1.03402	3.6860	8.0492	.74		
unidentified_Lachnospiraceae	1	6	4.8721	.27826	.11360	4.5800	5.1641	4.38		
	2	6	5.7757	1.07375	.43836	4.6489	6.9025	4.71		
	3	6	8.4657	1.52587	.62294	6.8644	10.0670	6.25		
	总计	18	6.3712	1.87439	.44180	5.4390	7.3033	4.38		
unclassified_Enterobacteriaceae	1	6	8.7320	5.13385	2.09588	3.3444	14.1196	3.46		
	2	6	1.4293	.51869	.21176	.8850	1.9737	.63		
	3	6	.2544	.07374	.03010	.1770	.3318	.20		
	总计	18	3.4719	4.76700	1.12359	1.1013	5.8425	.20		
Bifidobacterium	1	6	5.2063	1.85665	.75798	3.2579	7.1547	3.76		
	2	6	1.3278	.60879	.24854	.6889	1.9667	.50		
	3	6	1.7983	1.28726	.52552	.4474	3.1492	.34		
	总计	18	2.7774	2.18460	.51492	1.6911	3.8638	.34		
Oscillospira	1	6	.3682	.03739	.01527	.3290	.4074	.32		
	2	6	1.5182	1.24760	.50933	.2089	2.8275	.01		
	3	6	2.2324	.69499	.28373	1.5031	2.9618	1.24		
	总计	18	1.3729	1.10667	.26085	.8226	1.9233	.01		
Ruminococcus	1	6	.4724	.05697	.02326	.4126	.5322	.37		
	2	6	1.2249	.84326	.34426	.3399	2.1098	.42		
	3	6	2.8264	.84111	.34338	1.9437	3.7090	2.01		
	总计	18	1.5079	1.19934	.28269	.9114	2.1043	.37		
Dorea	1	6	.5464	.22472	.09174	.3106	.7822	.27		
	2	6	.8358	.35741	.14591	.4607	1.2109	.13		
	3	6	2.7823	.73539	.30022	2.0106	3.5541	1.80		
	总计	18	1.3882	1.12040	.26408	.8310	1.9453	.13		


ANOVA	
	¥­¤è©M	¦Û¥Ñ«×	§¡¤è	F	显µÛ©Ê	
unidentified_Ruminococcaceae	组间	422.095	2	211.048	12.165	.001	
	组内	260.228	15	17.349			
	总计	682.323	17				
unidentified_Bacteroidales	组间	251.400	2	125.700	68.468	.000	
	组内	27.539	15	1.836			
	总计	278.938	17				
unidentified_Clostridiales	组间	71.736	2	35.868	3.954	.042	
	组内	136.079	15	9.072			
	总计	207.815	17				
Blautia	组间	251.860	2	125.930	25.080	.000	
	组内	75.317	15	5.021			
	总计	327.176	17				
unidentified_Lachnospiraceae	组间	41.934	2	20.967	17.675	.000	
	组内	17.793	15	1.186			
	总计	59.727	17				
unclassified_Enterobacteriaceae	组间	253.159	2	126.579	14.259	.000	
	组内	133.154	15	8.877			
	总计	386.313	17				
Bifidobacterium	组间	53.758	2	26.879	14.729	.000	
	组内	27.374	15	1.825			
	总计	81.132	17				
Oscillospira	组间	10.616	2	5.308	7.802	.005	
	组内	10.205	15	.680			
	总计	20.820	17				
Ruminococcus	组间	17.344	2	8.672	18.298	.000	
	组内	7.109	15	.474			
	总计	24.453	17				
Dorea	组间	17.745	2	8.872	37.018	.000	
	组内	3.595	15	.240			
	总计	21.340	17				


¨Æ¦Z检验


¦h­«¤ñ较	
¦]变¶q	(I) VAR00001	(J) VAR00001	¥­§¡­È®t­È (I-J)	标­ã误®t	显µÛ©Ê	95% ¸m«H区间	
						¤U­­	¤W­­	
unidentified_Ruminococcaceae	LSD	1	2	8.40077*	2.40475	.003	3.2752	13.5264	
			3	-3.05179	2.40475	.224	-8.1774	2.0738	
		2	1	-8.40077*	2.40475	.003	-13.5264	-3.2752	
			3	-11.45256*	2.40475	.000	-16.5782	-6.3270	
		3	1	3.05179	2.40475	.224	-2.0738	8.1774	
			2	11.45256*	2.40475	.000	6.3270	16.5782	
unidentified_Bacteroidales	LSD	1	2	1.62640	.78228	.055	-.0410	3.2938	
			3	8.61487*	.78228	.000	6.9475	10.2823	
		2	1	-1.62640	.78228	.055	-3.2938	.0410	
			3	6.98848*	.78228	.000	5.3211	8.6559	
		3	1	-8.61487*	.78228	.000	-10.2823	-6.9475	
			2	-6.98848*	.78228	.000	-8.6559	-5.3211	
unidentified_Clostridiales	LSD	1	2	-1.78718	1.73896	.320	-5.4937	1.9193	
			3	-4.83547*	1.73896	.014	-8.5420	-1.1290	
		2	1	1.78718	1.73896	.320	-1.9193	5.4937	
			3	-3.04829	1.73896	.100	-6.7548	.6582	
		3	1	4.83547*	1.73896	.014	1.1290	8.5420	
			2	3.04829	1.73896	.100	-.6582	6.7548	
Blautia	LSD	1	2	-6.99254*	1.29372	.000	-9.7500	-4.2350	
			3	1.63142	1.29372	.227	-1.1261	4.3889	
		2	1	6.99254*	1.29372	.000	4.2350	9.7500	
			3	8.62396*	1.29372	.000	5.8665	11.3815	
		3	1	-1.63142	1.29372	.227	-4.3889	1.1261	
			2	-8.62396*	1.29372	.000	-11.3815	-5.8665	
unidentified_Lachnospiraceae	LSD	1	2	-.90366	.62881	.171	-2.2439	.4366	
			3	-3.59364*	.62881	.000	-4.9339	-2.2534	
		2	1	.90366	.62881	.171	-.4366	2.2439	
			3	-2.68998*	.62881	.001	-4.0303	-1.3497	
		3	1	3.59364*	.62881	.000	2.2534	4.9339	
			2	2.68998*	.62881	.001	1.3497	4.0303	
unclassified_Enterobacteriaceae	LSD	1	2	7.30266*	1.72017	.001	3.6362	10.9691	
			3	8.47761*	1.72017	.000	4.8112	12.1441	
		2	1	-7.30266*	1.72017	.001	-10.9691	-3.6362	
			3	1.17495	1.72017	.505	-2.4915	4.8414	
		3	1	-8.47761*	1.72017	.000	-12.1441	-4.8112	
			2	-1.17495	1.72017	.505	-4.8414	2.4915	
Bifidobacterium	LSD	1	2	3.87852*	.77994	.000	2.2161	5.5409	
			3	3.40804*	.77994	.001	1.7456	5.0705	
		2	1	-3.87852*	.77994	.000	-5.5409	-2.2161	
			3	-.47048	.77994	.555	-2.1329	1.1919	
		3	1	-3.40804*	.77994	.001	-5.0705	-1.7456	
			2	.47048	.77994	.555	-1.1919	2.1329	
Oscillospira	LSD	1	2	-1.14999*	.47620	.029	-2.1650	-.1350	
			3	-1.86422*	.47620	.001	-2.8792	-.8492	
		2	1	1.14999*	.47620	.029	.1350	2.1650	
			3	-.71423	.47620	.154	-1.7292	.3008	
		3	1	1.86422*	.47620	.001	.8492	2.8792	
			2	.71423	.47620	.154	-.3008	1.7292	
Ruminococcus	LSD	1	2	-.75245	.39746	.078	-1.5996	.0947	
			3	-2.35395*	.39746	.000	-3.2011	-1.5068	
		2	1	.75245	.39746	.078	-.0947	1.5996	
			3	-1.60150*	.39746	.001	-2.4487	-.7543	
		3	1	2.35395*	.39746	.000	1.5068	3.2011	
			2	1.60150*	.39746	.001	.7543	2.4487	
Dorea	LSD	1	2	-.28940	.28265	.322	-.8919	.3131	
			3	-2.23596*	.28265	.000	-2.8384	-1.6335	
		2	1	.28940	.28265	.322	-.3131	.8919	
			3	-1.94656*	.28265	.000	-2.5490	-1.3441	
		3	1	2.23596*	.28265	.000	1.6335	2.8384	
			2	1.94656*	.28265	.000	1.3441	2.5490	

*. ¥­§¡­È®t­Èªº显µÛ©Ê¤ô¥­为 0.05¡C	


齐©Ê¤l¶°


unidentified_Ruminococcaceae	
	VAR00001	个®×数	Alpha ªº¤l¶° = 0.05	
			1	2	
邓ªÖa	2	6	9.8652		
	1	6		18.2659	
	3	6		21.3177	
	显µÛ©Ê		1.000	.224	

将显¥Ü齐©Ê¤l¶°¤¤¦U个组ªº¥­§¡­È¡C	
a. ¨Ï¥Î调©M¥­§¡­È样¥»¤j¤p = 6.000¡C	


unidentified_Bacteroidales	
	VAR00001	个®×数	Alpha ªº¤l¶° = 0.05	
			1	2	
邓ªÖa	3	6	2.5023		
	2	6		9.4908	
	1	6		11.1172	
	显µÛ©Ê		1.000	.055	

将显¥Ü齐©Ê¤l¶°¤¤¦U个组ªº¥­§¡­È¡C	
a. ¨Ï¥Î调©M¥­§¡­È样¥»¤j¤p = 6.000¡C	


unidentified_Clostridiales	
	VAR00001	个®×数	Alpha ªº¤l¶° = 0.05	
			1	2	
邓ªÖa	1	6	8.6904		
	2	6	10.4776	10.4776	
	3	6		13.5259	
	显µÛ©Ê		.320	.100	

将显¥Ü齐©Ê¤l¶°¤¤¦U个组ªº¥­§¡­È¡C	
a. ¨Ï¥Î调©M¥­§¡­È样¥»¤j¤p = 6.000¡C	


Blautia	
	VAR00001	个®×数	Alpha ªº¤l¶° = 0.05	
			1	2	
邓ªÖa	3	6	2.4492		
	1	6	4.0806		
	2	6		11.0731	
	显µÛ©Ê		.227	1.000	

将显¥Ü齐©Ê¤l¶°¤¤¦U个组ªº¥­§¡­È¡C	
a. ¨Ï¥Î调©M¥­§¡­È样¥»¤j¤p = 6.000¡C	


unidentified_Lachnospiraceae	
	VAR00001	个®×数	Alpha ªº¤l¶° = 0.05	
			1	2	
邓ªÖa	1	6	4.8721		
	2	6	5.7757		
	3	6		8.4657	
	显µÛ©Ê		.171	1.000	

将显¥Ü齐©Ê¤l¶°¤¤¦U个组ªº¥­§¡­È¡C	
a. ¨Ï¥Î调©M¥­§¡­È样¥»¤j¤p = 6.000¡C	


unclassified_Enterobacteriaceae	
	VAR00001	个®×数	Alpha ªº¤l¶° = 0.05	
			1	2	
邓ªÖa	3	6	.2544		
	2	6	1.4293		
	1	6		8.7320	
	显µÛ©Ê		.505	1.000	

将显¥Ü齐©Ê¤l¶°¤¤¦U个组ªº¥­§¡­È¡C	
a. ¨Ï¥Î调©M¥­§¡­È样¥»¤j¤p = 6.000¡C	


Bifidobacterium	
	VAR00001	个®×数	Alpha ªº¤l¶° = 0.05	
			1	2	
邓ªÖa	2	6	1.3278		
	3	6	1.7983		
	1	6		5.2063	
	显µÛ©Ê		.555	1.000	

将显¥Ü齐©Ê¤l¶°¤¤¦U个组ªº¥­§¡­È¡C	
a. ¨Ï¥Î调©M¥­§¡­È样¥»¤j¤p = 6.000¡C	


Oscillospira	
	VAR00001	个®×数	Alpha ªº¤l¶° = 0.05	
			1	2	
邓ªÖa	1	6	.3682		
	2	6		1.5182	
	3	6		2.2324	
	显µÛ©Ê		1.000	.154	

将显¥Ü齐©Ê¤l¶°¤¤¦U个组ªº¥­§¡­È¡C	
a. ¨Ï¥Î调©M¥­§¡­È样¥»¤j¤p = 6.000¡C	


Ruminococcus	
	VAR00001	个®×数	Alpha ªº¤l¶° = 0.05	
			1	2	
邓ªÖa	1	6	.4724		
	2	6	1.2249		
	3	6		2.8264	
	显µÛ©Ê		.078	1.000	

将显¥Ü齐©Ê¤l¶°¤¤¦U个组ªº¥­§¡­È¡C	
a. ¨Ï¥Î调©M¥­§¡­È样¥»¤j¤p = 6.000¡C	


Dorea	
	VAR00001	个®×数	Alpha ªº¤l¶° = 0.05	
			1	2	
邓ªÖa	1	6	.5464		
	2	6	.8358		
	3	6		2.7823	
	显µÛ©Ê		.322	1.000	

将显¥Ü齐©Ê¤l¶°¤¤¦U个组ªº¥­§¡­È¡C	
a. ¨Ï¥Î调©M¥­§¡­È样¥»¤j¤p = 6.000¡C	


The genus level of rumen main microorganism


ONEWAY Prevotella Succiniclasticum Succinivibrio unidentified_Succinivibrionaceae
    unidentified_Bacteroidales Sharpea Clostridiales Ruminococcus unidentified_Lachnospiraceae
    Megasphaera BY VAR00001
  /STATISTICS DESCRIPTIVES
  /MISSING ANALYSIS
  /POSTHOC=DUNCAN LSD ALPHA(0.05).


单¦V


备ª`	
¤w创«Ø输¥X	10-JAN-2024 21:34:37	
ª`释		
输¤J	¬¡动数Õu¶°	数Õu¶°0	
	过滤¾¹	<无>	
	权­«	<无>	
	©î¤À¤å¥ó	<无>	
	¤u§@数Õu¤å¥ó¤¤ªº¦æ数	18	
¯Ê¥¢­È处²z	对¯Ê¥¢ªº©w义	将¥Î户©w义ªº¯Ê¥¢­È视为¯Ê¥¢¡C	
	¨Ï¥Îªº个®×数	¨C项¤ÀªRªº统计³£°ò¤_¨º¨Ç对¤_该¤ÀªR¤¤¨Ï¥Îªº¥ô¦ó变¶q³£¤£¨ã¦³¯Ê¥¢数Õuªº个®×¡C	
语ªk	ONEWAY Prevotella Succiniclasticum Succinivibrio unidentified_Succinivibrionaceae
    unidentified_Bacteroidales Sharpea Clostridiales Ruminococcus unidentified_Lachnospiraceae
    Megasphaera BY VAR00001
  /STATISTICS DESCRIPTIVES
  /MISSING ANALYSIS
  /POSTHOC=DUNCAN LSD ALPHA(0.05).	
资·½	处²zµ{§Ç时间	00:00:00.02	
	¯Ó¥Î时间	00:00:00.06	


[数Õu¶°0] 


´y­z	
	个®×数	¥­§¡­È	标­ã®t	标­ã误®t	¥­§¡­Èªº 95% ¸m«H区间	³Ì¤p­È		
					¤U­­	¤W­­			
Prevotella	1.00	6	21.2425	3.65472	1.49203	17.4071	25.0779	16.12		
	2.00	6	28.8863	3.92165	1.60101	24.7708	33.0018	21.28		
	3.00	6	34.4903	5.79744	2.36679	28.4062	40.5743	27.52		
	总计	18	28.2064	7.03941	1.65921	24.7057	31.7070	16.12		
Succiniclasticum	1.00	6	6.5232	2.10669	.86005	4.3124	8.7341	3.64		
	2.00	6	12.1536	4.51073	1.84150	7.4198	16.8873	4.11		
	3.00	6	25.0499	2.38481	.97359	22.5472	27.5526	20.89		
	总计	18	14.5756	8.52286	2.00886	10.3373	18.8139	3.64		
Succinivibrio	1.00	6	3.0380	1.88941	.77135	1.0552	5.0209	.36		
	2.00	6	15.8911	4.87811	1.99148	10.7718	21.0104	7.68		
	3.00	6	1.8476	.86902	.35478	.9356	2.7595	1.03		
	总计	18	6.9256	7.14672	1.68450	3.3716	10.4795	.36		
unidentified_Succinivibrionaceae	1.00	6	13.6389	5.42947	2.21657	7.9410	19.3367	9.00		
	2.00	6	.8803	.74507	.30417	.0984	1.6622	.11		
	3.00	6	1.1239	.47350	.19331	.6270	1.6208	.36		
	总计	18	5.2144	6.81788	1.60699	1.8239	8.6048	.11		
unidentified_Bacteroidales	1.00	6	10.8445	2.53844	1.03631	8.1806	13.5084	8.98		
	2.00	6	6.4590	2.33532	.95339	4.0083	8.9098	4.19		
	3.00	6	5.8694	.92337	.37696	4.9003	6.8384	4.08		
	总计	18	7.7243	2.99425	.70575	6.2353	9.2133	4.08		
Sharpea	1.00	6	3.0295	.87667	.35790	2.1095	3.9495	2.11		
	2.00	6	1.1080	.87502	.35723	.1897	2.0262	.11		
	3.00	6	3.0431	1.02386	.41799	1.9687	4.1176	1.69		
	总计	18	2.3935	1.27849	.30134	1.7578	3.0293	.11		
Clostridiales	1.00	6	1.8481	1.07759	.43993	.7172	2.9790	1.02		
	2.00	6	1.5088	.15286	.06241	1.3484	1.6692	1.30		
	3.00	6	2.4723	.93116	.38014	1.4951	3.4495	1.48		
	总计	18	1.9431	.87864	.20710	1.5061	2.3800	1.02		
Ruminococcus	1.00	6	1.6878	.28824	.11767	1.3853	1.9903	1.21		
	2.00	6	.6014	.35022	.14298	.2339	.9690	.14		
	3.00	6	1.4019	.71834	.29326	.6480	2.1557	.74		
	总计	18	1.2304	.66040	.15566	.9020	1.5588	.14		
unidentified_Lachnospiraceae	1.00	6	.5303	.25181	.10280	.2661	.7946	.24		
	2.00	6	.6882	.18201	.07430	.4972	.8792	.50		
	3.00	6	1.1931	.81554	.33294	.3372	2.0489	.54		
	总计	18	.8039	.55552	.13094	.5276	1.0801	.24		
Megasphaera	1.00	6	.9585	.37448	.15288	.5655	1.3515	.51		
	2.00	6	1.1785	.25479	.10402	.9111	1.4459	.82		
	3.00	6	.7195	.27499	.11227	.4309	1.0081	.45		
	总计	18	.9522	.34611	.08158	.7801	1.1243	.45		


ANOVA	
	¥­¤è©M	¦Û¥Ñ«×	§¡¤è	F	显µÛ©Ê	
Prevotella	组间	530.673	2	265.337	12.767	.001	
	组内	311.733	15	20.782			
	总计	842.406	17				
Succiniclasticum	组间	1082.505	2	541.253	53.287	.000	
	组内	152.361	15	10.157			
	总计	1234.866	17				
Succinivibrio	组间	727.680	2	363.840	38.815	.000	
	组内	140.605	15	9.374			
	总计	868.285	17				
unidentified_Succinivibrionaceae	组间	638.927	2	319.464	31.673	.000	
	组内	151.292	15	10.086			
	总计	790.220	17				
unidentified_Bacteroidales	组间	88.664	2	44.332	10.431	.001	
	组内	63.750	15	4.250			
	总计	152.414	17				
Sharpea	组间	14.875	2	7.437	8.640	.003	
	组内	12.913	15	.861			
	总计	27.787	17				
Clostridiales	组间	2.866	2	1.433	2.095	.158	
	组内	10.258	15	.684			
	总计	13.124	17				
Ruminococcus	组间	3.805	2	1.903	7.909	.005	
	组内	3.609	15	.241			
	总计	7.414	17				
unidentified_Lachnospiraceae	组间	1.438	2	.719	2.832	.090	
	组内	3.808	15	.254			
	总计	5.246	17				
Megasphaera	组间	.633	2	.316	3.379	.061	
	组内	1.404	15	.094			
	总计	2.036	17				


¨Æ¦Z检验


¦h­«¤ñ较	
¦]变¶q	(I) VAR00001	(J) VAR00001	¥­§¡­È®t­È (I-J)	标­ã误®t	显µÛ©Ê	95% ¸m«H区间	
						¤U­­	¤W­­	
Prevotella	LSD	1.00	2.00	-7.64380*	2.63200	.011	-13.2538	-2.0338	
			3.00	-13.24780*	2.63200	.000	-18.8578	-7.6378	
		2.00	1.00	7.64380*	2.63200	.011	2.0338	13.2538	
			3.00	-5.60400	2.63200	.050	-11.2140	.0060	
		3.00	1.00	13.24780*	2.63200	.000	7.6378	18.8578	
			2.00	5.60400	2.63200	.050	-.0060	11.2140	
Succiniclasticum	LSD	1.00	2.00	-5.63032*	1.84005	.008	-9.5523	-1.7083	
			3.00	-18.52665*	1.84005	.000	-22.4486	-14.6047	
		2.00	1.00	5.63032*	1.84005	.008	1.7083	9.5523	
			3.00	-12.89633*	1.84005	.000	-16.8183	-8.9744	
		3.00	1.00	18.52665*	1.84005	.000	14.6047	22.4486	
			2.00	12.89633*	1.84005	.000	8.9744	16.8183	
Succinivibrio	LSD	1.00	2.00	-12.85306*	1.76764	.000	-16.6207	-9.0854	
			3.00	1.19048	1.76764	.511	-2.5772	4.9581	
		2.00	1.00	12.85306*	1.76764	.000	9.0854	16.6207	
			3.00	14.04355*	1.76764	.000	10.2759	17.8112	
		3.00	1.00	-1.19048	1.76764	.511	-4.9581	2.5772	
			2.00	-14.04355*	1.76764	.000	-17.8112	-10.2759	
unidentified_Succinivibrionaceae	LSD	1.00	2.00	12.75857*	1.83359	.000	8.8504	16.6668	
			3.00	12.51492*	1.83359	.000	8.6067	16.4231	
		2.00	1.00	-12.75857*	1.83359	.000	-16.6668	-8.8504	
			3.00	-.24365	1.83359	.896	-4.1519	3.6646	
		3.00	1.00	-12.51492*	1.83359	.000	-16.4231	-8.6067	
			2.00	.24365	1.83359	.896	-3.6646	4.1519	
unidentified_Bacteroidales	LSD	1.00	2.00	4.38545*	1.19024	.002	1.8485	6.9224	
			3.00	4.97514*	1.19024	.001	2.4382	7.5121	
		2.00	1.00	-4.38545*	1.19024	.002	-6.9224	-1.8485	
			3.00	.58968	1.19024	.627	-1.9473	3.1266	
		3.00	1.00	-4.97514*	1.19024	.001	-7.5121	-2.4382	
			2.00	-.58968	1.19024	.627	-3.1266	1.9473	
Sharpea	LSD	1.00	2.00	1.92151*	.53567	.003	.7798	3.0633	
			3.00	-.01365	.53567	.980	-1.1554	1.1281	
		2.00	1.00	-1.92151*	.53567	.003	-3.0633	-.7798	
			3.00	-1.93516*	.53567	.003	-3.0769	-.7934	
		3.00	1.00	.01365	.53567	.980	-1.1281	1.1554	
			2.00	1.93516*	.53567	.003	.7934	3.0769	
Clostridiales	LSD	1.00	2.00	.33930	.47745	.488	-.6784	1.3570	
			3.00	-.62416	.47745	.211	-1.6418	.3935	
		2.00	1.00	-.33930	.47745	.488	-1.3570	.6784	
			3.00	-.96347	.47745	.062	-1.9811	.0542	
		3.00	1.00	.62416	.47745	.211	-.3935	1.6418	
			2.00	.96347	.47745	.062	-.0542	1.9811	
Ruminococcus	LSD	1.00	2.00	1.08639*	.28319	.002	.4828	1.6900	
			3.00	.28595	.28319	.329	-.3177	.8895	
		2.00	1.00	-1.08639*	.28319	.002	-1.6900	-.4828	
			3.00	-.80045*	.28319	.013	-1.4040	-.1968	
		3.00	1.00	-.28595	.28319	.329	-.8895	.3177	
			2.00	.80045*	.28319	.013	.1968	1.4040	
unidentified_Lachnospiraceae	LSD	1.00	2.00	-.15790	.29091	.595	-.7780	.4622	
			3.00	-.66271*	.29091	.038	-1.2828	-.0427	
		2.00	1.00	.15790	.29091	.595	-.4622	.7780	
			3.00	-.50481	.29091	.103	-1.1249	.1152	
		3.00	1.00	.66271*	.29091	.038	.0427	1.2828	
			2.00	.50481	.29091	.103	-.1152	1.1249	
Megasphaera	LSD	1.00	2.00	-.22002	.17663	.232	-.5965	.1564	
			3.00	.23903	.17663	.196	-.1374	.6155	
		2.00	1.00	.22002	.17663	.232	-.1564	.5965	
			3.00	.45906*	.17663	.020	.0826	.8355	
		3.00	1.00	-.23903	.17663	.196	-.6155	.1374	
			2.00	-.45906*	.17663	.020	-.8355	-.0826	

*. ¥­§¡­È®t­Èªº显µÛ©Ê¤ô¥­为 0.05¡C	


齐©Ê¤l¶°


Prevotella	
	VAR00001	个®×数	Alpha ªº¤l¶° = 0.05	
			1	2	
邓ªÖa	1.00	6	21.2425		
	2.00	6		28.8863	
	3.00	6		34.4903	
	显µÛ©Ê		1.000	.050	

将显¥Ü齐©Ê¤l¶°¤¤¦U个组ªº¥­§¡­È¡C	
a. ¨Ï¥Î调©M¥­§¡­È样¥»¤j¤p = 6.000¡C	


Succiniclasticum	
	VAR00001	个®×数	Alpha ªº¤l¶° = 0.05	
			1	2	3	
邓ªÖa	1.00	6	6.5232			
	2.00	6		12.1536		
	3.00	6			25.0499	
	显µÛ©Ê		1.000	1.000	1.000	

将显¥Ü齐©Ê¤l¶°¤¤¦U个组ªº¥­§¡­È¡C	
a. ¨Ï¥Î调©M¥­§¡­È样¥»¤j¤p = 6.000¡C	


Succinivibrio	
	VAR00001	个®×数	Alpha ªº¤l¶° = 0.05	
			1	2	
邓ªÖa	3.00	6	1.8476		
	1.00	6	3.0380		
	2.00	6		15.8911	
	显µÛ©Ê		.511	1.000	

将显¥Ü齐©Ê¤l¶°¤¤¦U个组ªº¥­§¡­È¡C	
a. ¨Ï¥Î调©M¥­§¡­È样¥»¤j¤p = 6.000¡C	


unidentified_Succinivibrionaceae	
	VAR00001	个®×数	Alpha ªº¤l¶° = 0.05	
			1	2	
邓ªÖa	2.00	6	.8803		
	3.00	6	1.1239		
	1.00	6		13.6389	
	显µÛ©Ê		.896	1.000	

将显¥Ü齐©Ê¤l¶°¤¤¦U个组ªº¥­§¡­È¡C	
a. ¨Ï¥Î调©M¥­§¡­È样¥»¤j¤p = 6.000¡C	


unidentified_Bacteroidales	
	VAR00001	个®×数	Alpha ªº¤l¶° = 0.05	
			1	2	
邓ªÖa	3.00	6	5.8694		
	2.00	6	6.4590		
	1.00	6		10.8445	
	显µÛ©Ê		.627	1.000	

将显¥Ü齐©Ê¤l¶°¤¤¦U个组ªº¥­§¡­È¡C	
a. ¨Ï¥Î调©M¥­§¡­È样¥»¤j¤p = 6.000¡C	


Sharpea	
	VAR00001	个®×数	Alpha ªº¤l¶° = 0.05	
			1	2	
邓ªÖa	2.00	6	1.1080		
	1.00	6		3.0295	
	3.00	6		3.0431	
	显µÛ©Ê		1.000	.980	

将显¥Ü齐©Ê¤l¶°¤¤¦U个组ªº¥­§¡­È¡C	
a. ¨Ï¥Î调©M¥­§¡­È样¥»¤j¤p = 6.000¡C	


Clostridiales	
	VAR00001	个®×数	Alpha ªº¤l¶° = 0.05	
			1	
邓ªÖa	2.00	6	1.5088	
	1.00	6	1.8481	
	3.00	6	2.4723	
	显µÛ©Ê		.074	

将显¥Ü齐©Ê¤l¶°¤¤¦U个组ªº¥­§¡­È¡C	
a. ¨Ï¥Î调©M¥­§¡­È样¥»¤j¤p = 6.000¡C	


Ruminococcus	
	VAR00001	个®×数	Alpha ªº¤l¶° = 0.05	
			1	2	
邓ªÖa	2.00	6	.6014		
	3.00	6		1.4019	
	1.00	6		1.6878	
	显µÛ©Ê		1.000	.329	

将显¥Ü齐©Ê¤l¶°¤¤¦U个组ªº¥­§¡­È¡C	
a. ¨Ï¥Î调©M¥­§¡­È样¥»¤j¤p = 6.000¡C	


unidentified_Lachnospiraceae	
	VAR00001	个®×数	Alpha ªº¤l¶° = 0.05	
			1	2	
邓ªÖa	1.00	6	.5303		
	2.00	6	.6882	.6882	
	3.00	6		1.1931	
	显µÛ©Ê		.595	.103	

将显¥Ü齐©Ê¤l¶°¤¤¦U个组ªº¥­§¡­È¡C	
a. ¨Ï¥Î调©M¥­§¡­È样¥»¤j¤p = 6.000¡C	


Megasphaera	
	VAR00001	个®×数	Alpha ªº¤l¶° = 0.05	
			1	2	
邓ªÖa	3.00	6	.7195		
	1.00	6	.9585	.9585	
	2.00	6		1.1785	
	显µÛ©Ê		.196	.232	

将显¥Ü齐©Ê¤l¶°¤¤¦U个组ªº¥­§¡­È¡C	
a. ¨Ï¥Î调©M¥­§¡­È样¥»¤j¤p = 6.000¡C	


The phylum level of intestinal main microorganism


ONEWAY Firmicutes Bacteroidetes Proteobacteria Actinobacteria Verrucomicrobia Tenericutes BY
    VAR00001
  /STATISTICS DESCRIPTIVES
  /MISSING ANALYSIS
  /POSTHOC=DUNCAN LSD ALPHA(0.05).


单¦V


备ª`	
¤w创«Ø输¥X	10-JAN-2024 20:41:00	
ª`释		
输¤J	¬¡动数Õu¶°	数Õu¶°0	
	过滤¾¹	<无>	
	权­«	<无>	
	©î¤À¤å¥ó	<无>	
	¤u§@数Õu¤å¥ó¤¤ªº¦æ数	18	
¯Ê¥¢­È处²z	对¯Ê¥¢ªº©w义	将¥Î户©w义ªº¯Ê¥¢­È视为¯Ê¥¢¡C	
	¨Ï¥Îªº个®×数	¨C项¤ÀªRªº统计³£°ò¤_¨º¨Ç对¤_该¤ÀªR¤¤¨Ï¥Îªº¥ô¦ó变¶q³£¤£¨ã¦³¯Ê¥¢数Õuªº个®×¡C	
语ªk	ONEWAY Firmicutes Bacteroidetes Proteobacteria Actinobacteria Verrucomicrobia Tenericutes BY
    VAR00001
  /STATISTICS DESCRIPTIVES
  /MISSING ANALYSIS
  /POSTHOC=DUNCAN LSD ALPHA(0.05).	
资·½	处²zµ{§Ç时间	00:00:00.02	
	¯Ó¥Î时间	00:00:00.03	


[数Õu¶°0] 


´y­z	
	个®×数	¥­§¡­È	标­ã®t	标­ã误®t	¥­§¡­Èªº 95% ¸m«H区间			
					¤U­­	¤W­­			
Firmicutes	1.00	6	54.8500	4.84587	1.97832	49.7646	59.9355			
	2.00	6	66.5856	12.38774	5.05727	53.5855	79.5858			
	3.00	6	71.3990	1.09701	.44785	70.2478	72.5502			
	总计	18	64.2782	10.17561	2.39841	59.2180	69.3384			
Bacteroidetes	1.00	6	17.5587	4.02621	1.64369	13.3334	21.7839			
	2.00	6	15.5893	4.41059	1.80062	10.9607	20.2179			
	3.00	6	18.1630	2.21473	.90416	15.8388	20.4872			
	总计	18	17.1037	3.63463	.85669	15.2962	18.9111			
Proteobacteria	1.00	6	9.4556	4.54190	1.85422	4.6891	14.2220			
	2.00	6	2.7984	1.14402	.46704	1.5978	3.9990			
	3.00	6	2.2043	.19657	.08025	1.9980	2.4106			
	总计	18	4.8194	4.23142	.99736	2.7152	6.9237			
Actinobacteria	1.00	6	5.0039	2.14655	.87632	2.7512	7.2565			
	2.00	6	3.9082	1.06714	.43566	2.7883	5.0281			
	3.00	6	2.4624	1.45634	.59455	.9341	3.9907			
	总计	18	3.7915	1.86037	.43849	2.8664	4.7166			
Verrucomicrobia	1.00	6	.0599	.01473	.00601	.0444	.0754			
	2.00	6	.5951	.25695	.10490	.3254	.8648			
	3.00	6	2.6496	1.13274	.46244	1.4609	3.8383			
	总计	18	1.1015	1.31003	.30878	.4501	1.7530			
Tenericutes	1.00	6	.4765	.16433	.06709	.3040	.6489			
	2.00	6	1.2450	.52306	.21354	.6961	1.7939			
	3.00	6	2.3248	.69396	.28331	1.5965	3.0530			
	总计	18	1.3488	.91575	.21584	.8934	1.8041			


ANOVA	
	¥­¤è©M	¦Û¥Ñ«×	§¡¤è	F	显µÛ©Ê	
Firmicutes	组间	869.522	2	434.761	7.322	.006	
	组内	890.710	15	59.381			
	总计	1760.232	17				
Bacteroidetes	组间	21.735	2	10.868	.804	.466	
	组内	202.843	15	13.523			
	总计	224.579	17				
Proteobacteria	组间	194.502	2	97.251	13.276	.000	
	组内	109.881	15	7.325			
	总计	304.383	17				
Actinobacteria	组间	19.499	2	9.750	3.718	.049	
	组内	39.337	15	2.622			
	总计	58.836	17				
Verrucomicrobia	组间	22.428	2	11.214	24.932	.000	
	组内	6.747	15	.450			
	总计	29.175	17				
Tenericutes	组间	10.345	2	5.173	19.840	.000	
	组内	3.911	15	.261			
	总计	14.256	17				


¨Æ¦Z检验


¦h­«¤ñ较	
¦]变¶q	(I) VAR00001	(J) VAR00001	¥­§¡­È®t­È (I-J)	标­ã误®t	显µÛ©Ê	95% ¸m«H区间	
						¤U­­	¤W­­	
Firmicutes	LSD	1.00	2.00	-11.73560*	4.44899	.019	-21.2184	-2.2528	
			3.00	-16.54896*	4.44899	.002	-26.0318	-7.0662	
		2.00	1.00	11.73560*	4.44899	.019	2.2528	21.2184	
			3.00	-4.81336	4.44899	.296	-14.2962	4.6694	
		3.00	1.00	16.54896*	4.44899	.002	7.0662	26.0318	
			2.00	4.81336	4.44899	.296	-4.6694	14.2962	
Bacteroidetes	LSD	1.00	2.00	1.96938	2.12312	.368	-2.5559	6.4947	
			3.00	-.60434	2.12312	.780	-5.1297	3.9210	
		2.00	1.00	-1.96938	2.12312	.368	-6.4947	2.5559	
			3.00	-2.57372	2.12312	.244	-7.0990	1.9516	
		3.00	1.00	.60434	2.12312	.780	-3.9210	5.1297	
			2.00	2.57372	2.12312	.244	-1.9516	7.0990	
Proteobacteria	LSD	1.00	2.00	6.65717*	1.56263	.001	3.3265	9.9878	
			3.00	7.25123*	1.56263	.000	3.9206	10.5819	
		2.00	1.00	-6.65717*	1.56263	.001	-9.9878	-3.3265	
			3.00	.59405	1.56263	.709	-2.7366	3.9247	
		3.00	1.00	-7.25123*	1.56263	.000	-10.5819	-3.9206	
			2.00	-.59405	1.56263	.709	-3.9247	2.7366	
Actinobacteria	LSD	1.00	2.00	1.09562	.93496	.260	-.8972	3.0884	
			3.00	2.54145*	.93496	.016	.5486	4.5343	
		2.00	1.00	-1.09562	.93496	.260	-3.0884	.8972	
			3.00	1.44583	.93496	.143	-.5470	3.4386	
		3.00	1.00	-2.54145*	.93496	.016	-4.5343	-.5486	
			2.00	-1.44583	.93496	.143	-3.4386	.5470	
Verrucomicrobia	LSD	1.00	2.00	-.53520	.38721	.187	-1.3605	.2901	
			3.00	-2.58971*	.38721	.000	-3.4150	-1.7644	
		2.00	1.00	.53520	.38721	.187	-.2901	1.3605	
			3.00	-2.05451*	.38721	.000	-2.8798	-1.2292	
		3.00	1.00	2.58971*	.38721	.000	1.7644	3.4150	
			2.00	2.05451*	.38721	.000	1.2292	2.8798	
Tenericutes	LSD	1.00	2.00	-.76849*	.29480	.020	-1.3968	-.1401	
			3.00	-1.84828*	.29480	.000	-2.4766	-1.2199	
		2.00	1.00	.76849*	.29480	.020	.1401	1.3968	
			3.00	-1.07979*	.29480	.002	-1.7081	-.4514	
		3.00	1.00	1.84828*	.29480	.000	1.2199	2.4766	
			2.00	1.07979*	.29480	.002	.4514	1.7081	

*. ¥­§¡­È®t­Èªº显µÛ©Ê¤ô¥­为 0.05¡C	


齐©Ê¤l¶°


Firmicutes	
	VAR00001	个®×数	Alpha ªº¤l¶° = 0.05	
			1	2	
邓ªÖa	1.00	6	54.8500		
	2.00	6		66.5856	
	3.00	6		71.3990	
	显µÛ©Ê		1.000	.296	

将显¥Ü齐©Ê¤l¶°¤¤¦U个组ªº¥­§¡­È¡C	
a. ¨Ï¥Î调©M¥­§¡­È样¥»¤j¤p = 6.000¡C	


Bacteroidetes	
	VAR00001	个®×数	Alpha ªº¤l¶° = 0.05	
			1	
邓ªÖa	2.00	6	15.5893	
	1.00	6	17.5587	
	3.00	6	18.1630	
	显µÛ©Ê		.268	

将显¥Ü齐©Ê¤l¶°¤¤¦U个组ªº¥­§¡­È¡C	
a. ¨Ï¥Î调©M¥­§¡­È样¥»¤j¤p = 6.000¡C	


Proteobacteria	
	VAR00001	个®×数	Alpha ªº¤l¶° = 0.05	
			1	2	
邓ªÖa	3.00	6	2.2043		
	2.00	6	2.7984		
	1.00	6		9.4556	
	显µÛ©Ê		.709	1.000	

将显¥Ü齐©Ê¤l¶°¤¤¦U个组ªº¥­§¡­È¡C	
a. ¨Ï¥Î调©M¥­§¡­È样¥»¤j¤p = 6.000¡C	


Actinobacteria	
	VAR00001	个®×数	Alpha ªº¤l¶° = 0.05	
			1	2	
邓ªÖa	3.00	6	2.4624		
	2.00	6	3.9082	3.9082	
	1.00	6		5.0039	
	显µÛ©Ê		.143	.260	

将显¥Ü齐©Ê¤l¶°¤¤¦U个组ªº¥­§¡­È¡C	
a. ¨Ï¥Î调©M¥­§¡­È样¥»¤j¤p = 6.000¡C	


Verrucomicrobia	
	VAR00001	个®×数	Alpha ªº¤l¶° = 0.05	
			1	2	
邓ªÖa	1.00	6	.0599		
	2.00	6	.5951		
	3.00	6		2.6496	
	显µÛ©Ê		.187	1.000	

将显¥Ü齐©Ê¤l¶°¤¤¦U个组ªº¥­§¡­È¡C	
a. ¨Ï¥Î调©M¥­§¡­È样¥»¤j¤p = 6.000¡C	


Tenericutes	
	VAR00001	个®×数	Alpha ªº¤l¶° = 0.05	
			1	2	3	
邓ªÖa	1.00	6	.4765			
	2.00	6		1.2450		
	3.00	6			2.3248	
	显µÛ©Ê		1.000	1.000	1.000	

将显¥Ü齐©Ê¤l¶°¤¤¦U个组ªº¥­§¡­È¡C	
a. ¨Ï¥Î调©M¥­§¡­È样¥»¤j¤p = 6.000¡C	


TNF-á£¬IFN-ã£¬IL-17£¬IL-10£¬IL-4£¬IgA


ONEWAY TNF£\ IFN£^ IL17 IL10 IL4 igA BY VAR00001
  /STATISTICS DESCRIPTIVES
  /MISSING ANALYSIS
  /POSTHOC=DUNCAN LSD ALPHA(0.05).


单¦V


备ª`	
¤w创«Ø输¥X	02-FEB-2024 13:04:12	
ª`释		
输¤J	¬¡动数Õu¶°	数Õu¶°0	
	过滤¾¹	<无>	
	权­«	<无>	
	©î¤À¤å¥ó	<无>	
	¤u§@数Õu¤å¥ó¤¤ªº¦æ数	21	
¯Ê¥¢­È处²z	对¯Ê¥¢ªº©w义	将¥Î户©w义ªº¯Ê¥¢­È视为¯Ê¥¢¡C	
	¨Ï¥Îªº个®×数	¨C项¤ÀªRªº统计³£°ò¤_¨º¨Ç对¤_该¤ÀªR¤¤¨Ï¥Îªº¥ô¦ó变¶q³£¤£¨ã¦³¯Ê¥¢数Õuªº个®×¡C	
语ªk	ONEWAY TNF£\ IFN£^ IL17 IL10 IL4 igA BY VAR00001
  /STATISTICS DESCRIPTIVES
  /MISSING ANALYSIS
  /POSTHOC=DUNCAN LSD ALPHA(0.05).	
资·½	处²zµ{§Ç时间	00:00:00.00	
	¯Ó¥Î时间	00:00:00.02	


[数Õu¶°0] 


´y­z	
	个®×数	¥­§¡­È	标­ã®t	标­ã误®t	¥­§¡­Èªº 95% ¸m«H区间			
					¤U­­	¤W­­			
TNF£\	1.00	7	375.7143	34.93191	13.20302	343.4077	408.0209			
	2.00	7	523.9286	116.52840	44.04360	416.1578	631.6994			
	3.00	7	763.0000	122.60412	46.34000	649.6101	876.3899			
	总计	21	554.2143	188.88911	41.21898	468.2330	640.1956			
IFN£^	1.00	7	138.0272	18.48664	6.98729	120.9299	155.1245			
	2.00	7	168.9796	23.64455	8.93680	147.1120	190.8472			
	3.00	7	191.4286	24.47483	9.25062	168.7931	214.0640			
	总计	21	166.1451	30.87423	6.73731	152.0913	180.1989			
IL17	1.00	7	40.7680	1.57840	.59658	39.3083	42.2278			
	2.00	7	43.6866	4.54535	1.71798	39.4829	47.8904			
	3.00	7	51.7512	5.22575	1.97515	46.9181	56.5842			
	总计	21	45.4019	6.14758	1.34151	42.6036	48.2003			
IL10	1.00	7	36.7485	1.25536	.47448	35.5875	37.9095			
	2.00	7	39.9851	6.78489	2.56445	33.7101	46.2601			
	3.00	7	42.8497	3.31943	1.25463	39.7797	45.9197			
	总计	21	39.8611	4.91031	1.07152	37.6260	42.0963			
IL4	1.00	7	12.9627	1.06257	.40161	11.9799	13.9454			
	2.00	7	16.5070	3.47066	1.31179	13.2972	19.7169			
	3.00	7	23.9746	4.98663	1.88477	19.3627	28.5864			
	总计	21	17.8148	5.79060	1.26361	15.1789	20.4506			
igA	1.00	7	104.1837	5.61997	2.12415	98.9861	109.3813			
	2.00	7	124.3003	15.22329	5.75386	110.2211	138.3795			
	3.00	7	182.0262	22.72339	8.58863	161.0106	203.0419			
	总计	21	136.8367	37.10555	8.09710	119.9465	153.7270			


ANOVA	
	¥­¤è©M	¦Û¥Ñ«×	§¡¤è	F	显µÛ©Ê	
TNF£\	组间	534596.643	2	267298.321	26.881	.000	
	组内	178985.268	18	9943.626			
	总计	713581.911	20				
IFN£^	组间	10065.328	2	5032.664	10.066	.001	
	组内	8999.028	18	499.946			
	总计	19064.356	20				
IL17	组间	453.094	2	226.547	13.469	.000	
	组内	302.760	18	16.820			
	总计	755.854	20				
IL10	组间	130.447	2	65.224	3.337	.059	
	组内	351.776	18	19.543			
	总计	482.223	20				
IL4	组间	442.374	2	221.187	17.443	.000	
	组内	228.246	18	12.680			
	总计	670.620	20				
igA	组间	22858.333	2	11429.166	43.976	.000	
	组内	4678.110	18	259.895			
	总计	27536.443	20				


¨Æ¦Z检验


¦h­«¤ñ较	
¦]变¶q	(I) VAR00001	(J) VAR00001	¥­§¡­È®t­È (I-J)	标­ã误®t	显µÛ©Ê	95% ¸m«H区间	
						¤U­­	¤W­­	
TNF£\	LSD	1.00	2.00	-148.21429*	53.30137	.012	-260.1963	-36.2323	
			3.00	-387.28571*	53.30137	.000	-499.2677	-275.3037	
		2.00	1.00	148.21429*	53.30137	.012	36.2323	260.1963	
			3.00	-239.07143*	53.30137	.000	-351.0535	-127.0894	
		3.00	1.00	387.28571*	53.30137	.000	275.3037	499.2677	
			2.00	239.07143*	53.30137	.000	127.0894	351.0535	
IFN£^	LSD	1.00	2.00	-30.95238*	11.95164	.018	-56.0618	-5.8429	
			3.00	-53.40136*	11.95164	.000	-78.5108	-28.2919	
		2.00	1.00	30.95238*	11.95164	.018	5.8429	56.0618	
			3.00	-22.44898	11.95164	.077	-47.5584	2.6605	
		3.00	1.00	53.40136*	11.95164	.000	28.2919	78.5108	
			2.00	22.44898	11.95164	.077	-2.6605	47.5584	
IL17	LSD	1.00	2.00	-2.91859	2.19219	.200	-7.5242	1.6870	
			3.00	-10.98310*	2.19219	.000	-15.5887	-6.3775	
		2.00	1.00	2.91859	2.19219	.200	-1.6870	7.5242	
			3.00	-8.06452*	2.19219	.002	-12.6701	-3.4589	
		3.00	1.00	10.98310*	2.19219	.000	6.3775	15.5887	
			2.00	8.06452*	2.19219	.002	3.4589	12.6701	
IL10	LSD	1.00	2.00	-3.23661	2.36299	.188	-8.2011	1.7279	
			3.00	-6.10119*	2.36299	.019	-11.0657	-1.1367	
		2.00	1.00	3.23661	2.36299	.188	-1.7279	8.2011	
			3.00	-2.86458	2.36299	.241	-7.8290	2.0999	
		3.00	1.00	6.10119*	2.36299	.019	1.1367	11.0657	
			2.00	2.86458	2.36299	.241	-2.0999	7.8290	
IL4	LSD	1.00	2.00	-3.54437	1.90341	.079	-7.5433	.4545	
			3.00	-11.01190*	1.90341	.000	-15.0108	-7.0130	
		2.00	1.00	3.54437	1.90341	.079	-.4545	7.5433	
			3.00	-7.46753*	1.90341	.001	-11.4664	-3.4686	
		3.00	1.00	11.01190*	1.90341	.000	7.0130	15.0108	
			2.00	7.46753*	1.90341	.001	3.4686	11.4664	
igA	LSD	1.00	2.00	-20.11662*	8.61718	.031	-38.2206	-2.0126	
			3.00	-77.84257*	8.61718	.000	-95.9466	-59.7386	
		2.00	1.00	20.11662*	8.61718	.031	2.0126	38.2206	
			3.00	-57.72595*	8.61718	.000	-75.8300	-39.6219	
		3.00	1.00	77.84257*	8.61718	.000	59.7386	95.9466	
			2.00	57.72595*	8.61718	.000	39.6219	75.8300	

*. ¥­§¡­È®t­Èªº显µÛ©Ê¤ô¥­为 0.05¡C	


齐©Ê¤l¶°


TNF£\	
	VAR00001	个®×数	Alpha ªº¤l¶° = 0.05	
			1	2	3	
邓ªÖa	1.00	7	375.7143			
	2.00	7		523.9286		
	3.00	7			763.0000	
	显µÛ©Ê		1.000	1.000	1.000	

将显¥Ü齐©Ê¤l¶°¤¤¦U个组ªº¥­§¡­È¡C	
a. ¨Ï¥Î调©M¥­§¡­È样¥»¤j¤p = 7.000¡C	


IFN£^	
	VAR00001	个®×数	Alpha ªº¤l¶° = 0.05	
			1	2	
邓ªÖa	1.00	7	138.0272		
	2.00	7		168.9796	
	3.00	7		191.4286	
	显µÛ©Ê		1.000	.077	

将显¥Ü齐©Ê¤l¶°¤¤¦U个组ªº¥­§¡­È¡C	
a. ¨Ï¥Î调©M¥­§¡­È样¥»¤j¤p = 7.000¡C	


IL17	
	VAR00001	个®×数	Alpha ªº¤l¶° = 0.05	
			1	2	
邓ªÖa	1.00	7	40.7680		
	2.00	7	43.6866		
	3.00	7		51.7512	
	显µÛ©Ê		.200	1.000	

将显¥Ü齐©Ê¤l¶°¤¤¦U个组ªº¥­§¡­È¡C	
a. ¨Ï¥Î调©M¥­§¡­È样¥»¤j¤p = 7.000¡C	


IL10	
	VAR00001	个®×数	Alpha ªº¤l¶° = 0.05	
			1	2	
邓ªÖa	1.00	7	36.7485		
	2.00	7	39.9851	39.9851	
	3.00	7		42.8497	
	显µÛ©Ê		.188	.241	

将显¥Ü齐©Ê¤l¶°¤¤¦U个组ªº¥­§¡­È¡C	
a. ¨Ï¥Î调©M¥­§¡­È样¥»¤j¤p = 7.000¡C	


IL4	
	VAR00001	个®×数	Alpha ªº¤l¶° = 0.05	
			1	2	
邓ªÖa	1.00	7	12.9627		
	2.00	7	16.5070		
	3.00	7		23.9746	
	显µÛ©Ê		.079	1.000	

将显¥Ü齐©Ê¤l¶°¤¤¦U个组ªº¥­§¡­È¡C	
a. ¨Ï¥Î调©M¥­§¡­È样¥»¤j¤p = 7.000¡C	


igA	
	VAR00001	个®×数	Alpha ªº¤l¶° = 0.05	
			1	2	3	
邓ªÖa	1.00	7	104.1837			
	2.00	7		124.3003		
	3.00	7			182.0262	
	显µÛ©Ê		1.000	1.000	1.000	

将显¥Ü齐©Ê¤l¶°¤¤¦U个组ªº¥­§¡­È¡C	
a. ¨Ï¥Î调©M¥­§¡­È样¥»¤j¤p = 7.000¡C	
